# Action observation perspective influences the effectiveness of combined action observation and motor imagery training for novices learning an Osoto Gari judo throw

**DOI:** 10.1038/s41598-024-70315-8

**Published:** 2024-08-28

**Authors:** Samantha Chye, Ashika Chembila Valappil, Ryan Knight, Andrew Greene, David Shearer, Cornelia Frank, Ceri Diss, Adam Bruton

**Affiliations:** 1 School of Life and Health Sciences, University of Roehampton, London, UK; 2 Faculty of Life Sciences and Education, University of South Wales, Cardiff, UK; 3 Department of Sports and Movement Science, Osnabrück University, Osnabrück, Germany; 4 Department of Life Sciences, Brunel University London, Uxbridge, UB8 3PH UK

**Keywords:** Motor imagery during action observation, Action simulation, Movement kinematics, Mental representation, Self-efficacy, Human behaviour, Psychology

## Abstract

Combined action observation and motor imagery (AOMI) training improves motor skill performance, but limited research has investigated possible moderating factors for this intervention. This study examined the influence of action observation (AO) perspective on the effectiveness of AOMI training for novices learning a ‘shadow’ Osoto Gari judo throw. Thirty novice participants were randomly assigned to AOMI training that displayed egocentric footage (AOMI_EGO_) or allocentric footage (AOMI_ALLO_) of the Osoto Gari, or Control training. A motor learning design incorporating pre-test (Day 1), acquisition (Days 2–6), post-test (Day 7), and retention-test (Day 14) was adopted. Motor skill performance, self-efficacy, and mental representation structures were recorded as measures of learning. There were mixed effects for motor skill performance across the three training conditions utilized in this study, with AOMI_ALLO_ training significantly reducing error scores for final right hip flexion angle and peak right ankle velocity compared to AOMI_EGO_ training. Self-efficacy increased for all training conditions over time. Both AOMI_EGO_ and AOMI_ALLO_ training led to improved functional changes in mental representation structures over time compared to Control training. The findings suggest AOMI training led to improved perceptual-cognitive scaffolding, irrespective of AO perspective, and offer some support for the use of AOMI_ALLO_ training to facilitate novice learning of complex, serial motor skills in sport.

## Introduction

Action simulation refers to the internal representation of motor programs without overt movement, and is an umbrella term covering the use of action observation (AO), motor imagery (MI), and combined action observation and motor imagery (AOMI)^1^. AO training involves the deliberate and structured observation of oneself or another performing the target movement(s)^2^. In contexts such as sport, AO training benefits motor skill performance and learning (e.g.,^3^) through refining working memory processes (e.g.,^4^) and enhancing motivational factors such as self-efficacy (e.g.,^5^). MI training involves the internal generation of visual and kinesthetic imagery that is involved in movement execution^6^. The efficacy of MI training is widely supported in sport, with similar benefits in behavioral (for meta-analyses, see Refs.^7,8^), psychological (e.g., Ref.^9^) and cognitive outcomes (e.g., Ref.^4^) as those reported for AO training. Given the effectiveness of both AO and MI training, researchers have studied AOMI across the last decade (see Ref.^10^ for initial literature review). AOMI training involves a person repetitively and systematically observing a target movement and simultaneously imagining the physiological sensations and kinesthetic experiences associated with that movement^11^.

Across the last decade, three theoretical accounts have been proposed to explain the processes underlying possible movement benefits associated with AOMI training. The first two propositions, the Dual-Action Simulation Hypothesis [DASH; Ref.^11^] and Visual Guidance Hypothesis [VGH; Ref.^12^] provide different neuroscientific accounts for such benefits. The DASH proposes that a person will generate separate motor representations for the observed and imagined actions and maintain these as two parallel sensorimotor streams in the brain when they engage in AOMI. The VGH argues that the observed action serves as a visual primer for the imagined action during AOMI, strengthening the motor representation generated for the imagined action. Both the DASH and VGH imply that AOMI training, if repeated, will lead to greater improvements in motor skill learning due to increased activation in motor regions of the brain when compared to independent AO or MI^13^.

More recently, Frank et al.^14–16^ drew from the Cognitive Action Architecture Approach (CAA-A) to postulate about the effects of AOMI on motor learning from a cognitive psychology perspective. According to the CAA-A framework, AOMI facilitates learning of a motor skill because it links cognitive representations to perceptual ones, refining the mental representations that guide movement execution (i.e., perceptual-cognitive scaffolding). More generally speaking from an ideomotor point of view, action effects are anticipated during learning via AOMI, leading to the structuring of (quasi)action effects (i.e. perceptual-cognitive scaffolding^16^) towards a more appropriate mental representation, which then guides future action. AO and MI reportedly influence mental representations through different mechanisms. Specifically, AO develops sequencing and timing aspects of the mental representation through visual presentation of movement information (i.e. actual action effects), and MI develops sensory aspects of mental representations through the cognitive generation of visual and kinesthetic aspects of movement (i.e., quasi action effects^16^). Through combining observed and imagined actions, each contributing to perceptual-cognitive scaffolding, it is proposed that AOMI develops mental representations of action in the long-term memory more comprehensively than either AO or MI independently, leading to more effective movement execution^14,17^.

From a neurophysiological perspective, AO and MI activate overlapping but distinct neural regions in the brain^18^. This suggests AOMI may lead to increased activity in shared regions for AO and MI (i.e., premotor and rostral parietal) and result in more widespread activity across distinct brain areas associated with AO (i.e., small areas of the inferior frontal gyrus and inferior/superior parietal lobule) and MI (i.e., SMA, PMd, PMv, DLPFC). Current neuroscientific evidence using a range of modalities supports this notion, as cortico-motor activity is increased during AOMI of an action compared to independent AO or MI of that same action (e.g., Refs.^19–21^). A recent meta-analysis^22^ reported that corticospinal excitability was facilitated for AOMI compared to AO and control conditions, but not MI conditions. This increased activation of the motor system suggests that AOMI training has the potential to improve movement execution via plastic-like changes in the motor system, in a similar manner to physical practice^23^.

AOMI training leads to improved movement outcomes when compared to Control and AO training^22^. Studies have found that prolonged bouts of AOMI training benefits postural control^24^ and muscle force production (e.g., Ref.^25^) compared to Control training, and dart throwing accuracy (e.g., Ref.^26^), and movement kinematics (e.g., Ref.^27^) compared to Control and AO training. While the findings from these studies support the short-term physical benefits attainable through AOMI training, they do not discern possible longer-term benefits (i.e., the learning effects associated with AOMI training). Motor learning can be defined as “a set of [internal] processes associated with practice or experience leading to relatively permanent gains in the capability for skilled performance” (Ref.^28^, p.51). This suggests improved motor skill performance immediately after the completion of training is not a reliable indicator of learning. Retention tests are commonly employed in the motor learning literature to assess longer-term changes in motor skill performance, but few studies to-date have utilized delayed assessments of performance changes after AOMI training interventions in healthy populations (see e.g., Refs.^29,30^). In clinical populations, AOMI training has also been shown to facilitate longer-term improvements in performance (see e.g., Refs.^31,32^). All of these studies demonstrated prolonged benefits in movement outcomes during the retention test, providing early evidence that AOMI training may facilitate learning as well as performance of a motor skill.

To date, AOMI training studies have had high heterogeneity and few studies have directly investigated potential moderating factors when attempting to improve motor skill performance via this intervention^22^. Indeed, to advance the field, it has been recommended that the efficacy of AOMI training be robustly examined across different contexts and motor skill types, and that intervention design considerations be rigorously tested^17^. Despite its popularity in applied sciences, sport has received relatively little research attention when studying the effects of AOMI training on motor skill performance (*n* = 4 studies [25%])^22^. Across contexts, most studies have explored the effects of AOMI training on simple fine (*n* = 12 studies [75%]) and/or discrete (*n* = 14 studies, [88%]) motor skills such as dart throwing and golf putting in sport^22^. The effectiveness of AOMI training may be more variable for novice compared to skilled learners, especially for serial or continuous motor skills that hold greater complexity^33^. In partial disagreement with this proposition, improved performance has been reported for highly complex serial motor skills after both AO^34^ and MI training interventions^35^. This suggests that AOMI training may also be effective for novice performance and learning of complex motor skills in sport.

The perspective adopted when delivering the AO component of the AOMI training intervention provides a noteworthy intervention design consideration. Research investigating perspective in MI-only interventions has demonstrated task-dependent benefits for external imagery over internal^36,37^, however, these differences are less clear for AO perspective. It was noted that eight studies (50%) included in a recent meta-analysis exploring the effects of AOMI training on movement outcomes^22^ adopted an egocentric AO perspective (AOMI_EGO_; viewing the action through their own eyes as though they were performing it) and eight studies (50%) adopted an allocentric AO perspective (AOMI_ALLO_; viewing the action as though another person was performing it). Benefits in motor skill performance have been reported for both AOMI_EGO_ (e.g., Refs.^26,27,38^) and AOMI_ALLO_ training (e.g., Refs.^25,39^). Indeed, Chye et al.^22^ reported no difference in the effectiveness of AOMI training based on AO perspective when synthesizing the data from different populations and types of motor skill. It is possible that adopting different AO perspectives during AOMI training may benefit motor skill performance and learning through varying mechanisms. AOMI_EGO_ provides a viewpoint that closely matches movement execution and likely benefits motor skill performance and learning by facilitating kinesthetic imagery ability during AOMI training^40,41^. This approach aligns with the proposal that MI drives the increased activity in motor regions of the brain and subsequent movement benefits that may be associated with AOMI training^12^. However, AOMI_EGO_ provides limited visual information about the movement, potentially negating any movement benefits associated with the AO component of the AOMI training (see Refs.^3,34^ for syntheses of AO literature). AOMI_ALLO_ provides more visual information depicting movement kinematics than AOMI_EGO_ and likely benefits motor skill performance and learning by increasing the learners understanding of key aspects of the movement^11^. AOMI_ALLO_ will typically prioritise the AO component of the intervention, potentially reducing any benefits gained from the MI component as this requires mental rotation of the movement displayed through the AO component^23^.

**Figure 1:**
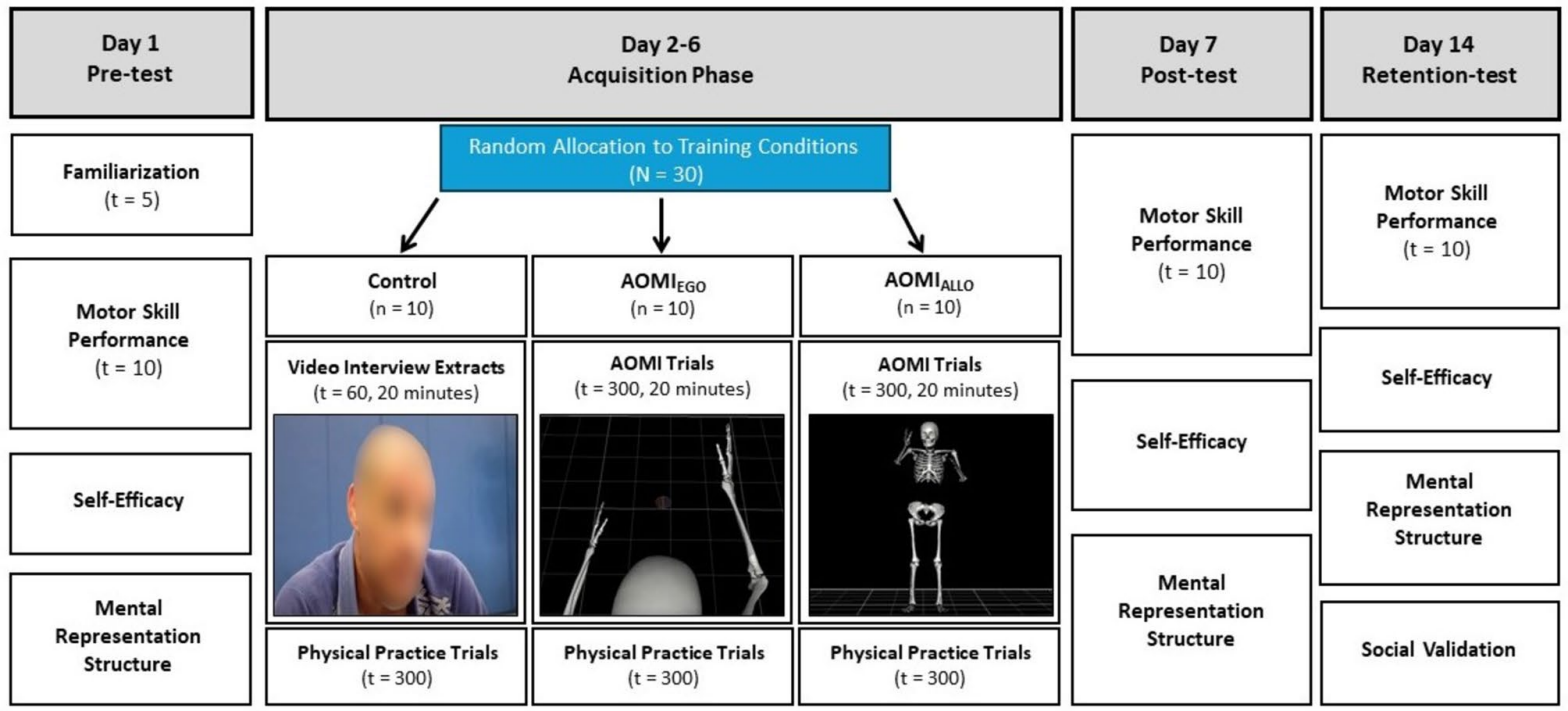
Experimental procedures across all training conditions*.* On Day 1, participants became familiar with the target movement before completing a pre-test. During acquisition across Days 2–6, all participants engaged in a total of 20-min non-physical practice based on their allocated training condition alongside 300 trials of physical practice of the shadow Osoto Gari movement without an opponent. On Day 7, participants returned to the laboratory for a second time and completed a post-test. On Day 14, participants returned to the laboratory for a third time and completed a retention-test followed by a social validation questionnaire and semi-structured interview.

In the present study (see Fig. 1 for an overview of the study protocol), we aimed to examine the influence of AO perspective on the effectiveness of AOMI training for novices learning a complex motor skill in sport. Specifically, we compared novices learning of an Osoto Gari judo throw after five days of AOMI_EGO_, AOMI_ALLO_, or Control training. Learning was inferred by recording biomechanical kinematic markers underpinning successful movements as a measure of motor skill performance, task-specific self-efficacy through a self-report questionnaire, and mental representation structures using structural dimensional analysis of mental representation (SDA-M)^42^. All three outcome measures were recorded immediately pre- and post-intervention, as well as after a one-week retention period.

Based on the theoretical perspectives of the DASH^11^, VGH^12^, and CAA-A frameworks^14–16^, it was predicted that both AOMI training conditions would have positive effects on the learning measures compared to the Control training condition. As there is reportedly no difference in the effectiveness of AOMI training based on AO perspective^22^ and studies have reported movement outcome benefits for both AOMI_EGO_ (e.g., Refs.^27,38^) and AOMI_ALLO_ training (e.g., Refs.^25,39^), it was hypothesized that there would be no differences between AOMI_EGO_ and AOMI_ALLO_ training conditions in terms of effects on the learning measures.

## Results

### Motor skill performance

The Multi-Level Linear Model (MLM) indicated that random effect residuals for ‘participant’ accounted for a significant portion of the variance across all kinematic variables as well as self-efficacy scores, supporting the decision to model these random effects (see Table S1 in supplementary results section for additional detail).

#### Initial and final right hip flexion

**Figure 2:**
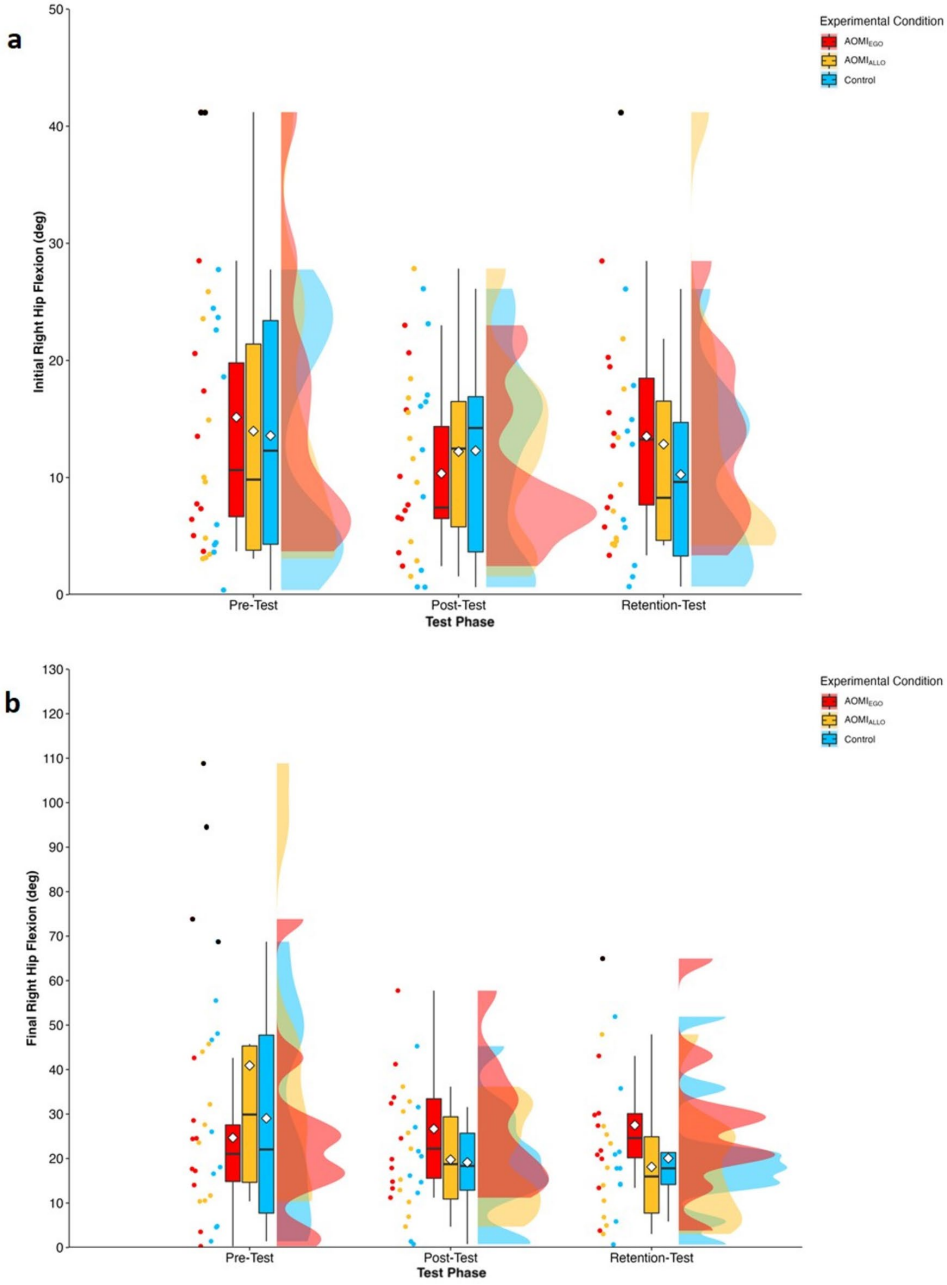
Box and violin plots with raw data points displaying error scores for (**a**) initial hip flexion angle and (**b**) final hip flexion angle for AOMI_EGO_ (red), AOMI_ALLO_ (yellow), and Control (green) training conditions across the three test phases. Thick horizontal black lines represent the median average and white diamonds represent the mean average for each box plot. Individual participant error scores are represented by circular markers, with blacked out markers representing data points that were omitted through outlier diagnostics.

There was no significant main effect of test phase (*F* [2, 56.94] = 0.12, *p* = 0.89), training condition (*F* [2, 29.35] = 0.02, *p* = 0.98) or interaction effect between training condition and test phase (*F* [4, 56.93] = 0.91, *p* = 0.46) for initial right hip flexion error scores (Fig. 2a). There was no significant main effect of test phase (*F* [2, 53.59] = 1.87, *p* = 0.16), no significant main effect of training condition (*F* [2, 27.84] = 0.15, *p* = 0.86), and an interaction effect that approached significance between training condition and test phase (*F* [4, 53.57] = 2.49, *p* = 0.05) for final right hip flexion error scores. Follow-up pairwise comparisons (Fig. 2b) for the near-significant interaction effect suggested that final right hip flexion error scores significantly reduced between pre- and retention-test for the AOMI_ALLO_ training condition (*p* = 0.03), but did not change for the Control or AOMI_EGO_ training conditions across the test phases (*p* > 0.05).

#### Base of support

**Figure 3:**
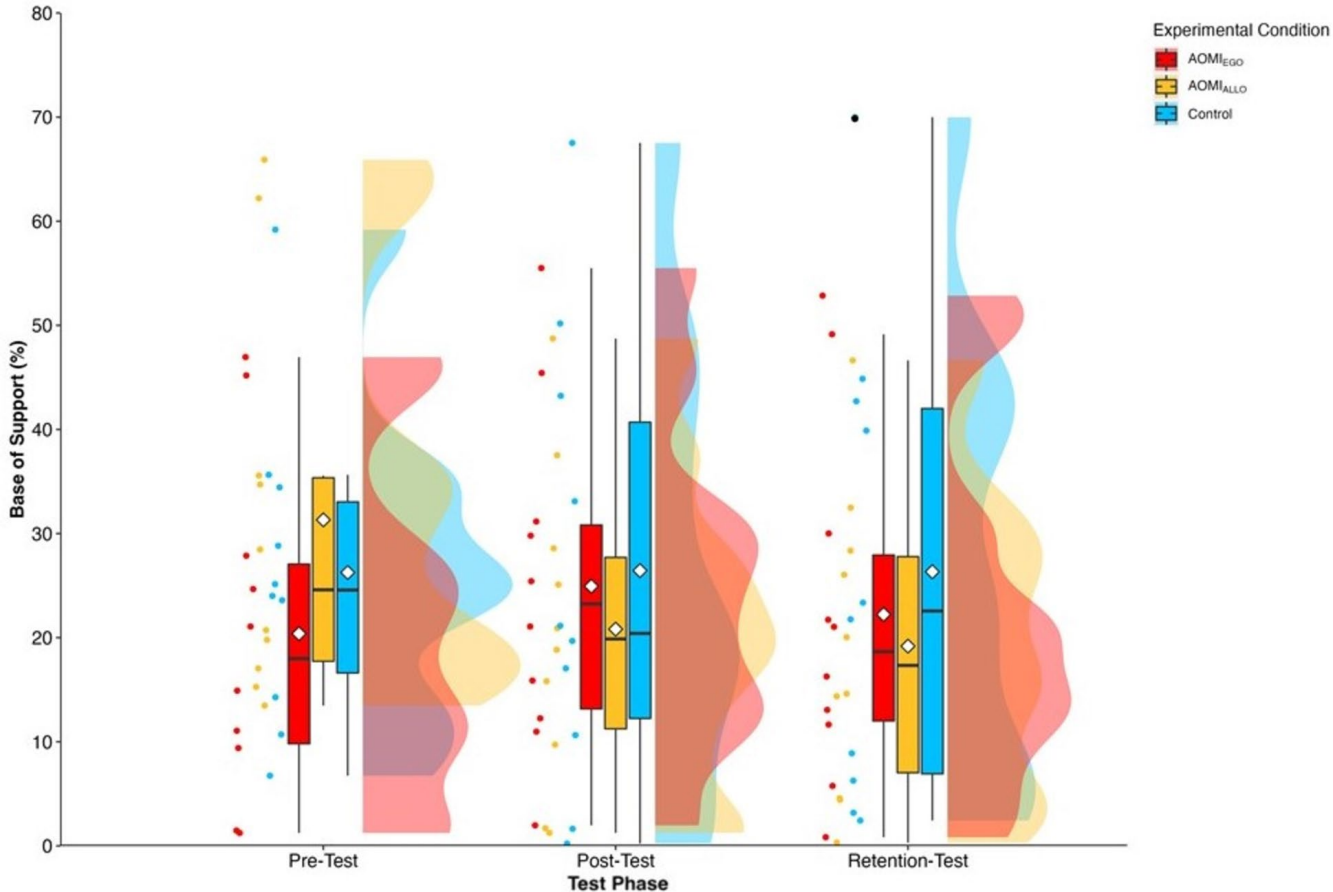
Box and violin plots with raw data points displaying base of support error scores for AOMI_EGO_ (red), AOMI_ALLO_ (yellow), and Control (green) training conditions across the three test phases. Thick horizontal black lines represent the median average and white diamonds represent the mean average for each box plot. Individual participant error scores are represented by circular markers, with blacked out markers representing data points that were omitted through outlier diagnostics.

There was no significant main effect of test phase (*F* [2, 58.22] = 1.08, *p* = 0.35), training condition (*F* [2, 29.1] = 0.13, *p* = 0.88) or interaction effect between training condition and test phase (*F* [4, 58.22] = 1.75, *p* = 0.15) for base of support error scores (Fig. 3).

#### Horizontal and vertical centre of mass

**Figure 4:**
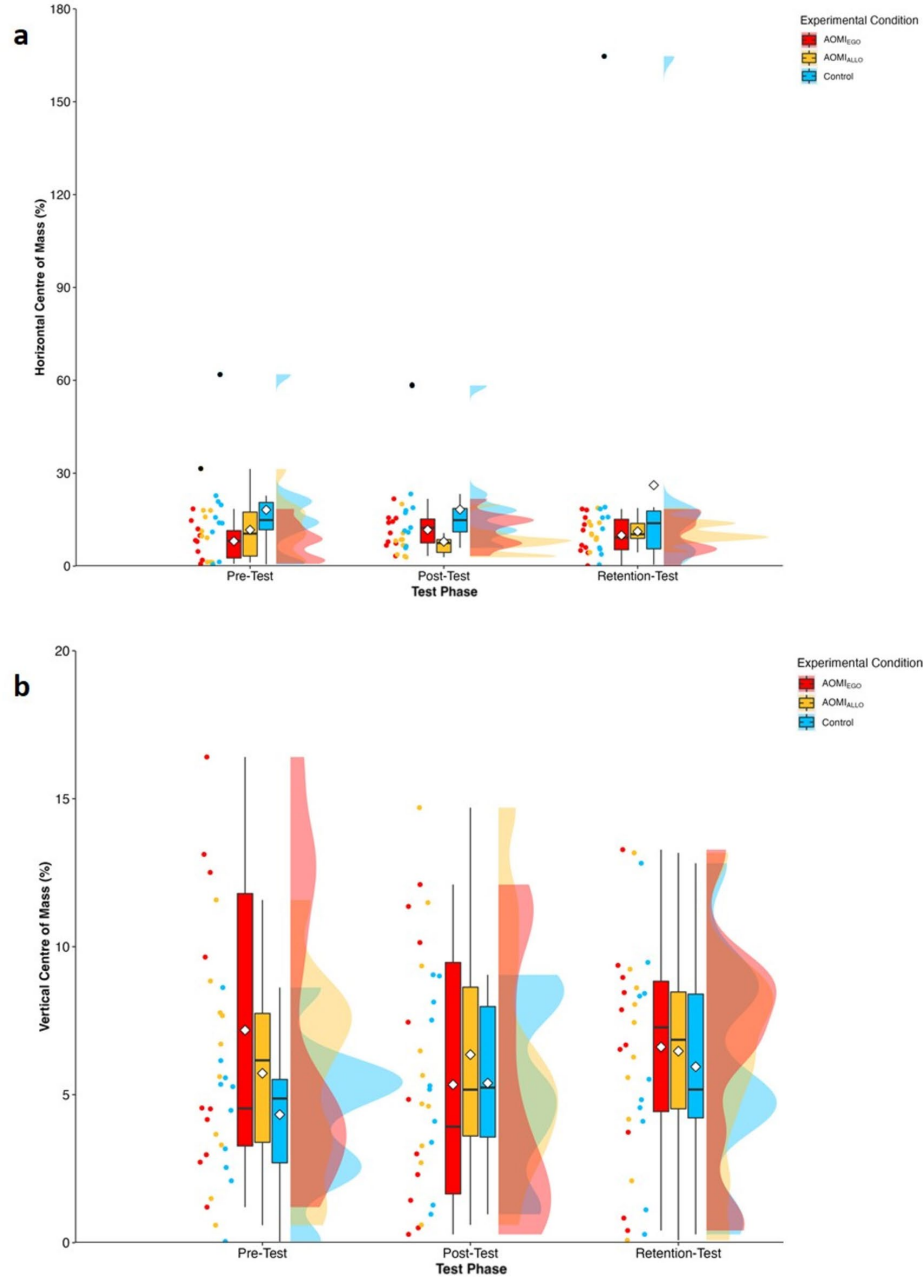
Box and violin plots with raw data points displaying error scores for (**a**) horizontal centre of mass and (**b**) vertical centre of mass for AOMI_EGO_ (red), AOMI_ALLO_ (yellow), and Control (green) training conditions across the three test phases. Thick horizontal black lines represent the median average and white diamonds represent the mean average for each box plot. Individual participant error scores are represented by circular markers, with blacked out markers representing data points that were omitted through outlier diagnostics.

There was no significant main effect of test phase (*F* [2, 57.32] = 0.38, *p* = 0.68), or training condition (*F* [2, 29.13] = 1.18, *p* = 0.32), but the interaction effect between training condition and test phase approached significance (*F* [4, 57.31] = 2.53, *p* = 0.05) for horizontal centre of mass error scores. Follow-up pairwise comparisons (Fig. 4a) for the near-significant interaction effect reported no changes over time across the three training conditions (*p* > 0.05). There was no significant main effect of test phase (*F* [2, 60] = 0.75, *p* = 0.48), training condition (*F* [2, 30] = 0.37, *p* = 0.70) or interaction effect between training condition and test phase (*F* [4, 60] = 1.29, *p* = 0.29) for vertical centre of mass error scores (Fig. 4b).

#### Peak right ankle velocity, peak right shoulder velocity, and peak velocity time difference

**Figure 5:**
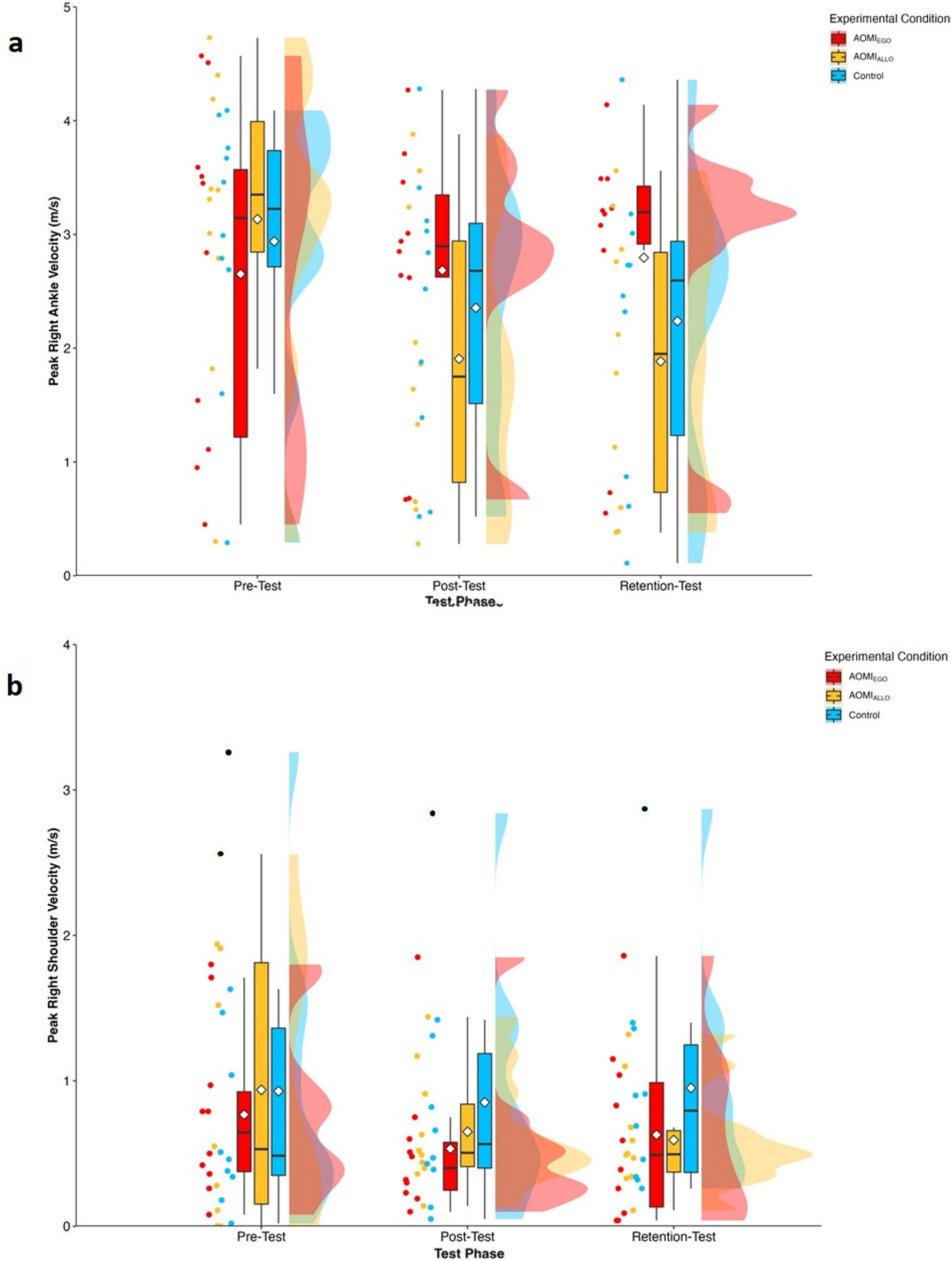
Box and violin plots with raw data points displaying error scores for (**a**) peak right ankle velocity and (**b**) peak right shoulder velocity for AOMI_EGO_ (red), AOMI_ALLO_ (yellow), and Control (green) training conditions across the three test phases. Thick horizontal black lines represent the median average and white diamonds represent the mean average for each box plot. Individual participant error scores are represented by circular markers, with blacked out markers representing data points that were omitted through outlier diagnostics.

**Figure 6:**
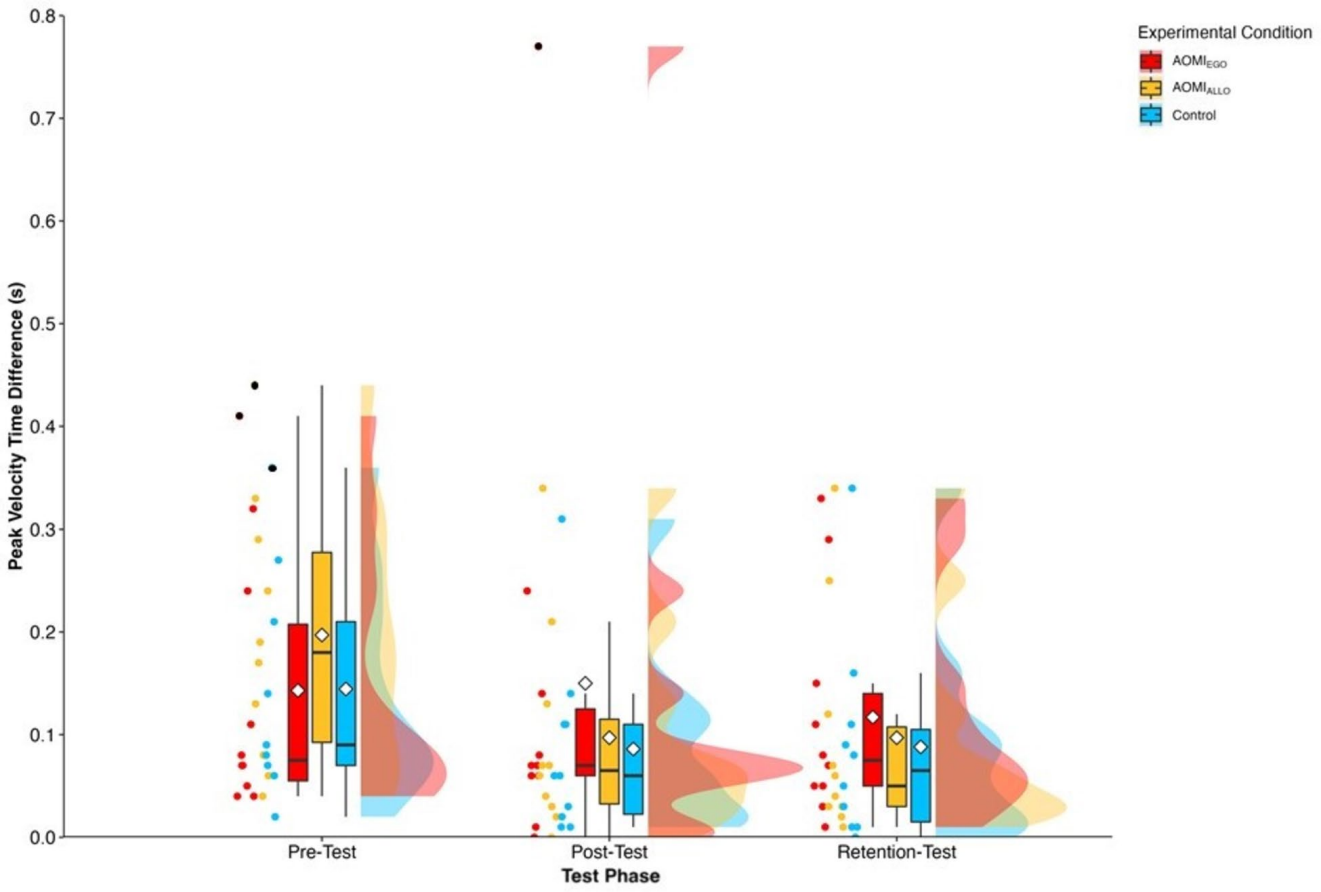
Box and violin plots with raw data points displaying peak velocity time difference error scores for AOMI_EGO_ (red), AOMI_ALLO_ (yellow), and Control (green) training conditions across the three test phases. Thick horizontal black lines represent the median average and white diamonds represent the mean average for each box plot. Individual participant error scores are represented by circular markers, with blacked out markers representing data points that were omitted through outlier diagnostics.

There was a significant main effect of test phase (*F* [2, 60] = 10.74, *p* < 0.001), no significant main effect of training condition (*F* [2, 30] = 0.33, *p* = 0.72) and a significant interaction effect between training condition and test phase (*F* [4, 60] = 4.53, *p* = 0.003) for peak right ankle velocity error scores. Follow-up pairwise comparisons (Fig. 5a) for the interaction effect between training condition and test phase suggested that peak right ankle velocity error scores significantly reduced across time for the AOMI_ALLO_ training condition (pre vs post, *p* < 0.001; pre vs retention, *p* < 0.01) and Control training condition (pre vs post, *p* = 0.049; pre vs retention, *p* = 0.02), but did not change for the AOMI_EGO_ training condition (pre vs post, *p* = 0.98; pre vs retention, *p* = 0.88). There was no significant main effect of test phase (*F* [2, 57.17] = 1.52, *p* = 0.23), training condition (*F* [2, 29] = 0.02, *p* = 0.98) or interaction effect between training condition and test phase (*F* [4, 57.16] = 0.65, *p* = 0.63) for peak right shoulder velocity error scores (Fig. 5b). There was a significant main effect of test phase (*F* [2, 53.25] = 4.94, *p* = 0.01), but no significant main effect of training condition (*F* [2, 27.26] = 0.07, *p* = 0.93) or interaction effect between training condition and test phase (*F* [4, 53.23] = 0.57, *p* = 0.68) for peak velocity time difference error scores. Follow-up pairwise comparisons (Fig. 6) for the main effect of test phase suggested that peak velocity time difference error scores significantly reduced between pre-test and post-test (*ß* = − 0.05, t_(62.7)_ = 2.80, *p* = 0.02), but did not significantly differ between pre-test and retention-test (*ß* = 0.04, t_(63.4)_ = 2.32, *p* = 0.06), or between post-test and retention-test (*ß* = − 0.01, t_(62.5)_ = − 0.51, *p* = 0.87).

### Self-efficacy

**Figure 7:**
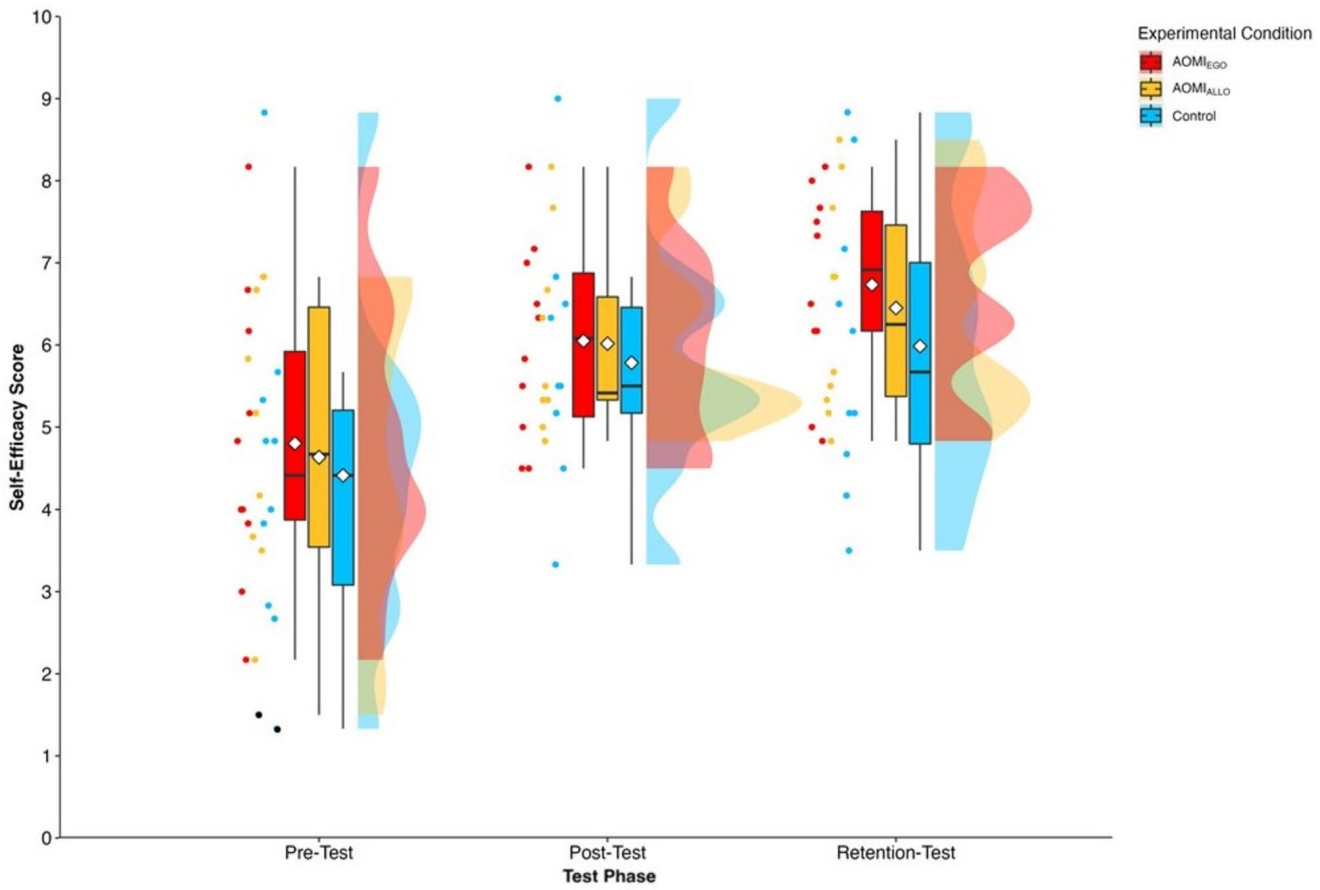
Box and violin plots with raw data points displaying self-efficacy scores for AOMI_EGO_ (red), AOMI_ALLO_ (yellow), and Control (green) training conditions across the three test phases. Thick horizontal black lines represent the median average and white diamonds represent the mean average for each box plot. Individual participant error scores are represented by circular markers, with blacked out markers representing data points that were omitted through outlier diagnostics.

There was a significant main effect of test phase (*F* [2, 58.13] = 32.32, *p* < 0.001), but no significant main effect of training condition (*F* [2, 29.93] = 0.31, *p* = 0.74) or interaction effect between training condition and test phase (*F* [4, 58.13] = 0.4, *p* = 0.81) for self-efficacy scores. Follow-up pairwise comparisons (Fig. 7) for the main effect of test phase suggested that self-efficacy scores significantly increased between pre-test and post-test (*ß* = − 1.15, t_(48)_ = − 4.75, *p* < 0.001) and pre-test and retention-test (*ß* = − 1.48, t_(47.5)_ = − 6.12, *p* < 0.001), but did not significantly differ between post-test and retention-test (*ß* = − 0.33, t_(46.7)_ = − 1.68, *p* = 0.22).

### Mental representation structure

#### Egocentric AOMI (AOMI_EGO_) training condition

**Figure 8:**
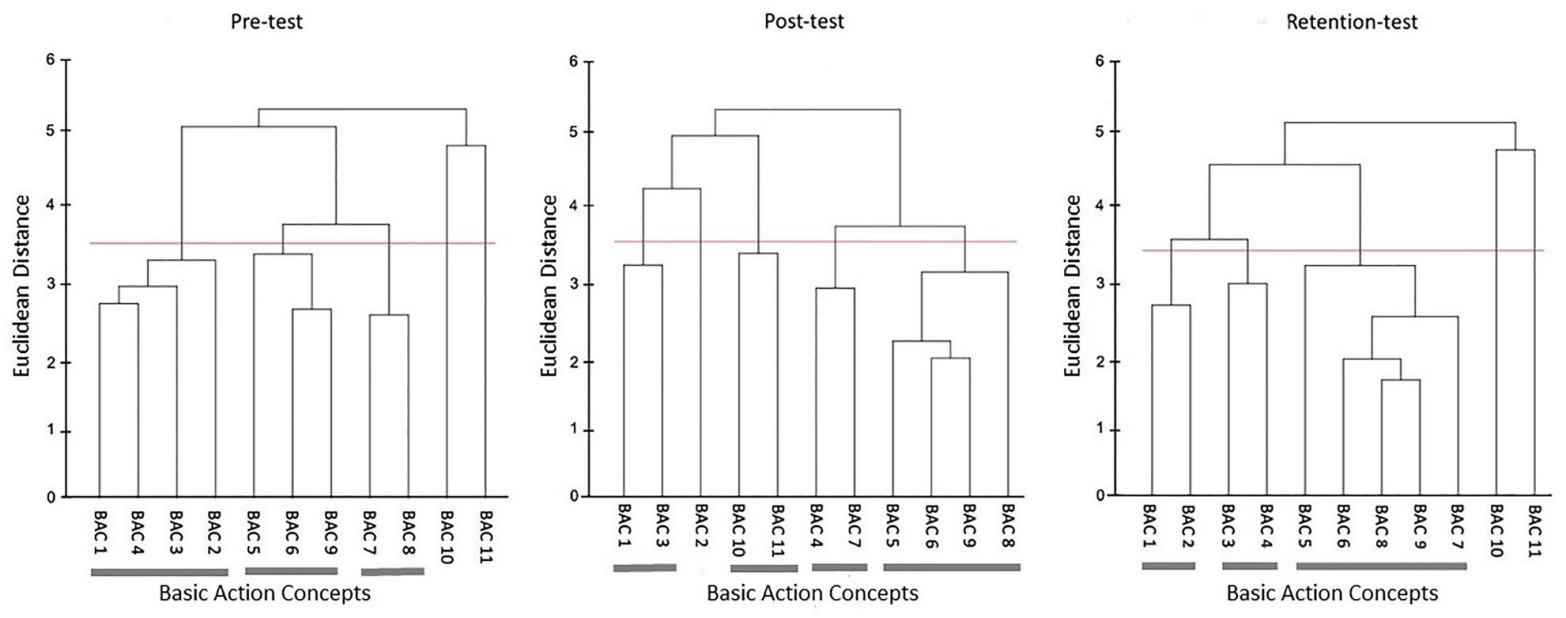
Mean group tree diagram of the Osoto Gari for the AOMI_EGO_ training condition across the three test phases. Each BAC is labelled on the x-axis (for the list of BACs, see Table 2). The numbers on the y-axis display Euclidean distances. The lower the Euclidean distance between BACs, the closer the BACs are. The horizontal red line marks the critical value *d*_*crit*_ for a given *α*-level (*d*_*crit*_ = 3.41; *α* = 0.05). Thick horizontal grey lines below the BAC labels on the x-axis depict clusters of BACs.

The mean group tree diagrams (Fig. 8) for participants allocated to the AOMI_EGO_ training condition comprised of three clusters at pre-test (BACs [1 4 3 2]; [5 6 9]; [7 8]), four clusters at post-test (BACs [1 3]; [10 11]; [4 7]; [5 6 9 8]), and three clusters at retention-test (BACs [1 2]; [3 4]; [5 6 8 9 7]). Analysis of invariance revealed that the representation structure for participants allocated to the AOMI_EGO_ training condition was variant between pre-test and post-test (*λ* = 0.43), pre-test and retention test (*λ* = 0.59), and post-test and retention test (*λ* = 0.44). Mental representation structures became more like the model structure over time between pre-test and post-test (*ARI*_*pre*_ = 0.47, *ARI*_*post*_ = 0.62, *ARI*_*diff*_ =  + 0.15), pre-test and retention-test (*ARI*_*pre*_ = 0.47, *ARI*_*retention* =_ 0.80, *ARI*_*diff*_ =  + 0.33), and post-test and retention-test (*ARI*_*post*_ = 0.62, *ARI*_*retention* =_ 0.80, *ARI*_*diff*_ =  + 0.18).

#### Allocentric AOMI (AOMI_ALLO_) training condition

**Figure 9:**
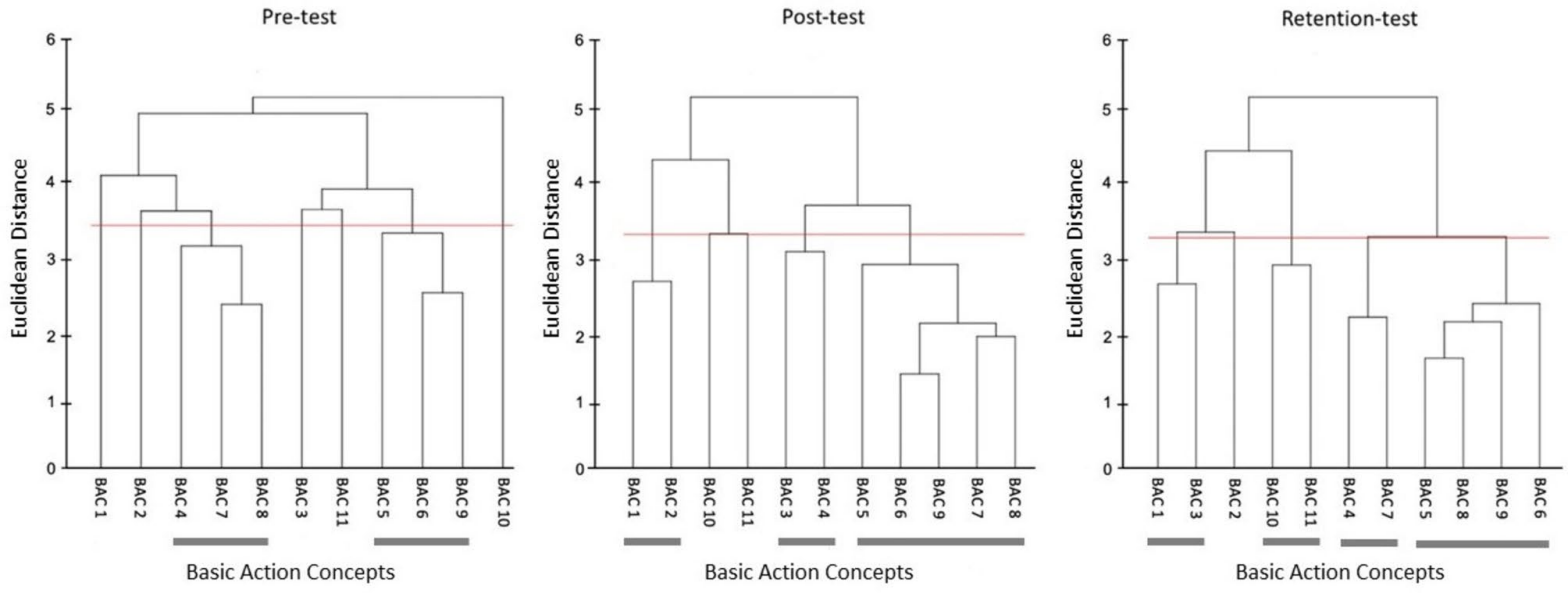
Mean group tree diagram of the Osoto Gari for the AOMI_ALLO_ training condition across the three test phases. Each BAC is labelled on the x-axis (for the list of BACs, see Table 2). The numbers on the y-axis display Euclidean distances. The lower the Euclidean distance between BACs, the closer the BACs are. The horizontal red line marks the critical value *d*_*crit*_ for a given *α*-level (*d*_*crit*_ = 3.41; *α* = 0.05). Thick horizontal grey lines below the BAC labels on the x-axis depict clusters of BACs.

The mean group tree diagrams (Fig. 9) for participants allocated to the AOMI_ALLO_ training condition comprised of two clusters at pre-test (BACs [4 7 8]; [5 6 9]), three clusters at post-test (BACs [1 2]; [3 4]; [5 6 9 7 8]), and four clusters at retention-test (BACs [1 3]; [10 11]; [4 7]; [5 8 9 6]). Analysis of invariance revealed that the mental representation structure for participants allocated to the AOMI_ALLO_ training condition was variant between pre-test and post-test (*λ* = 0.48), pre-test and retention test (*λ* = 0.37), and post-test and retention test (*λ* = 0.44). Mental representation structures became more like the reference structure over time between pre-test and post-test (*ARI*_*pre*_ = 0.29, *ARI*_*post*_ = 0.80, *ARI*_*diff*_ =  + 0.51) and pre-test and retention-test (*ARI*_*pre*_ = 0.29, *ARI*_*retention*_ = 0.62, *ARI*_*diff*_ =  + 0.33), but became less like the reference structure between post-test and retention-test (*ARI*_*post*_ = 0.80, *ARI*_*retention*_ = 0.62, *ARI*_*diff*_ = − 0.18).

#### Control training condition

**Figure 10:**
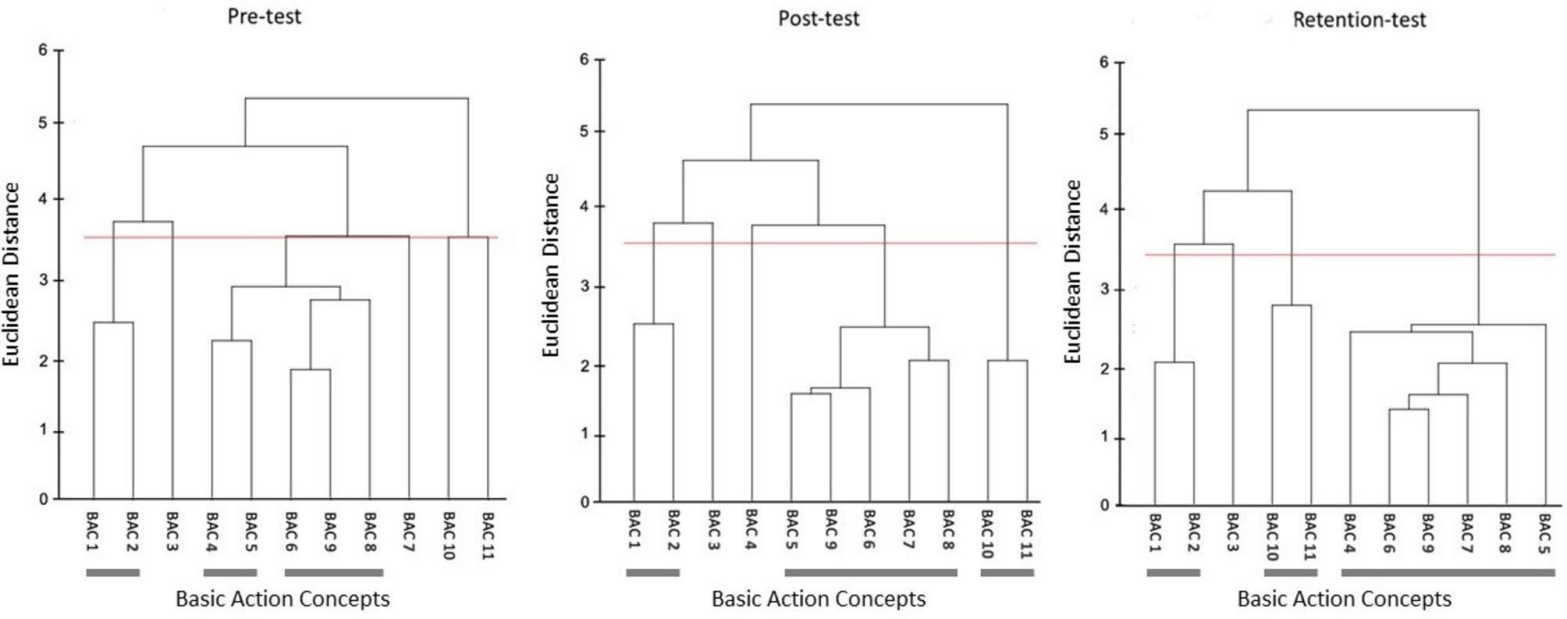
Mean group tree diagram of the Osoto Gari for the Control training condition across the three test phases. Each BAC is labelled on the x-axis (for the list of BACs, see Table 2). The numbers on the y-axis display Euclidean distances. The lower the Euclidean distance between BACs, the closer the BACs are. The horizontal red line marks the critical value *d*_*crit*_ for a given *α*-level (*d*_*crit*_ = 3.41; *α* = 0.05). Thick horizontal grey lines below the BAC labels on the x-axis depict clusters of BACs.

The mean group tree diagrams (Fig. 10) for participants allocated to the Control training condition comprised of three clusters at pre-test (BACs [1 2]; [4 5]; [6 9 8]), post-test (BACs [1 2]; [5 9 6 7 8]; [10 11]), and retention-test (BACs [1 2]; [10 11]; [4 6 9 7 8 5]). Analysis of invariance revealed that the mental representation structure for participants allocated to the Control training condition was variant between both pre-test and post-test (*λ* = 0.47), and pre-test and retention-test (*λ* = 0.49), but invariant between post-test and retention-test (*λ* = 0.69). Mental representation structures became more like the reference structure over time between pre-test and post-test (*ARI*_*pre*_ = 0.43, *ARI*_*post*_ = 0.90, *ARI*_*diff*_ =  + 0.47) and pre-test and retention-test (*ARI*_*pre*_ = 0.43, *ARI*_*retention*_ = 0.69, *ARI*_*diff*_ =  + 0.26), but became less like the reference structure between post-test and retention-test (*ARI*_*post*_ = 0.90, *ARI*_*retention*_ = 0.69, *ARI*_*diff*_ = − 0.21).

## Discussion

This study investigated the influence of AO perspective on the effectiveness of AOMI training for novices learning an Osoto Gari judo throw by comparing the effects of AOMI_EGO_ and AOMI_ALLO_ training on motor skill performance, self-efficacy and mental representation structures. It was hypothesized that both AOMI training conditions would lead to improvements across these learning measures when compared to Control training. The results of this study partly supported these predictions. Self-efficacy scores increased across the three test phases for all training conditions, with AOMI_EGO_ training causing the descriptively largest increase between pre- and retention-test. Mental representation structures became more functional after all training conditions, with larger improvements reported between pre- and retention-test for the two AOMI training. There were mixed effects for motor skill performance across the three training conditions utilized in this study, with AOMI_ALLO_ training significantly reducing error scores for final right hip flexion angle and peak right ankle velocity compared to AOMI_EGO_ training.

Our results provide limited support for the hypothesis that both AOMI training conditions would benefit motor skill performance to a greater extent than the Control training condition due to a lack of significant differences in error score changes for most kinematic measures between training conditions. This finding opposes recent meta-analytical findings showing that AOMI training incorporating physical practice has a small to moderate positive effect on performance of different types of motor skill compared to Control training^22^. However, it is worth nothing that the majority of motor skills included in Chye and colleagues’^22^ meta-analysis were classified as simple and discrete tasks, which could potentially explain the difference in findings for this study as the novices were tasked with learning a complex serial motor skill. These are not the first null or mixed effects published for AOMI training (e.g., Refs.^27,43^) and might also be partly explained by the lack of feedback available to the novices during training sessions. In training, participants performed the Osoto Gari throw without an opponent, reducing the tactile and kinesthetic feedback available to the novice learners. The quantity and quality of sensory feedback, alongside the performer’s ability to integrate feedback, influences improvement in motor skill performance resulting from simulation interventions^33^. Indeed, research has demonstrated that feedback has informational and motivational properties that can benefit motor learning (e.g., Ref.^44^). In the context of this study, the reduced sensory feedback during execution of the Osoto Gari throw combined with the lack of augmented feedback after physical trials might have disrupted any benefits to motor skill performance and learning attained by participants after AOMI training.

Despite the mixed results for motor skill performance, AOMI_ALLO_ training led to reduced error scores across a greater proportion (5/8, 63%) of the eight kinematic measures of motor skill performance than AOMI_EGO_ training (3/8, 38%), and significantly reduced error scores for final right hip flexion angle compared to both AOMI_EGO_ and Control training, and peak right ankle velocity compared to AOMI_EGO_ training. These findings provide some support for the influence of AO perspective when using AOMI training to facilitate novice learning of the Osoto Gari, a complex, serial motor skill in sport. Both of these discrete kinematic measures relate to the right leg swing back phase that occurs sequentially late in the movement. This is interesting because this movement information is not displayed in the egocentric footage incorporated during AOMI_EGO_ training due to the leg swing occurring behind the head position of the avatar depicting the skilled model’s performance of the Osoto Gari throw. This suggests that the AO component of AOMI training can benefit aspects of movement execution by conveying useful visual information related to movement technique and force production^23^, rather than just providing a primer for the generation of kinesthetic information for use with the MI component of AOMI training^12^.

It is likely that the ideal AO perspective for AOMI training will vary based on the target motor skill and the proficiency of the learner or performer^17^. This sentiment aligns with the claims of McNeill and colleagues^33^ that novice learners typically benefit more from AOMI than independent AO or MI because it provides them with a combination of visual and sensory information that facilitates the internal representation of the movement and reinforces the learning process. It is possible that the use of multiple perspectives, as well as self- and other-models, could benefit motor skill performance beyond the use of a single AO perspective or model as it will facilitate error detection/correction processes^29^, and provide additional augmented feedback that is known to benefit movement outcomes^33^.

Self-efficacy scores increased across the three test phases for all training conditions, but contrary to our hypothesis, there were no significant differences in self-efficacy changes between AOMI and Control training. The AOMI training conditions were predicted to increase self-efficacy to a greater extent than Control training because AO^3^ and MI^9^ training independently enhances efficacy beliefs, and thus it is feasible that combining these approaches through AOMI training would have an additive effect. This proposal draws support from recent suggestions that AO and MI training increase efficacy beliefs through the provision of two different antecedents of efficacy, namely vicarious and mastery experiences^17,45^. It is possible that AOMI_EGO_ training resulted in the descriptively largest increase in self-efficacy beliefs as it more directly targeted enactive mastery experiences, which are the strongest source of self-efficacy^45^. Specifically, AOMI_EGO_ is proposed to facilitate the imagery component during AOMI training^40,41^, reinforcing the recent performance accomplishments attained by the novice learners when physically performing the motor skill during the test and acquisition phases of this study. However, the lack of significant differences between self-efficacy change scores for the different training conditions suggests novices lacked the necessary expertise with the movement task to facilitate their imagery sufficiently during AOMI training. It is likely that the novice learners predominantly derived their self-efficacy beliefs from physical practice of the Osoto Gari throw due to a lack of previous performance accomplishments. Vicarious experiences can represent an important source of efficacy information for novice learners due to their lack of previous experience with a motor skill^46^. However, the use of a skilled model for the AO component of the AOMI training may have caused the novice learners to compare themselves unfavourably with the model due to the large disparities in their ability to perform the shadow Osoto Gari, resulting in negative vicarious experiences and minimizing the efficacy improvements attained via AOMI training (e.g., Ref.^29^).

Mental representation structures became more functional across all training conditions, with larger improvements reported between pre- and retention-test for the two AOMI training conditions. This supports our hypothesis and aligns with previous research showing that, when combined with physical practice, action simulation training leads to greater functional changes in novices’ mental representations of a motor skill compared to physical practice alone (e.g., Refs.^29,47^). The lack of differences in changes in mental representation structure between AOMI_EGO_ and AOMI_ALLO_ training conditions could be explained by the two AO perspectives developing mental representations through different mechanisms^17^, while both leading to functional changes through perceptual-cognitive scaffolding^14–16^. As indicated in the social validation data (see Supplementary file), AOMI_EGO_ training was perceived to benefit understanding of the Osoto Gari throw by facilitating the novice learners’ imagery of the movement, which is likely to develop components of the mental representations related to the kinesthetic feedback associated with movement execution. Alternatively, AOMI_ALLO_ training provides greater visual information regarding technical aspects of the Osoto Gari throw, suggesting it might develop components of the mental representation related to sequencing and timing of the different movement components.

The disconnect between the findings for mental representation structure and motor skill performance in this study suggests perceptual-cognitive scaffolding occurs prior to motor learning through engagement in AOMI training^14–16^. Both the AOMI training groups showed greater functional changes in their mental representation structures, but this did not lead to robust improvements in motor skill performance based on the discrete kinematic measures collected, as shown in previous MI and AOMI training studies^29,42^. This indicates that an adaptation to perceptual-cognitive prerequisites of action occur at an early stage of learning through movement simulation^48^, whereby these mental representations are updated based on action effects that are anticipated during this process, leading to structuring of (quasi) action effects. However, while this updating of structures guides future action, it appears this does not immediately translate into improved motor learning for complex motor skills such as the movement task adopted in this study when a novice learner engages in AOMI training^29,42^. A large body of research has shown that expert athletes hold a refined mental representation structure compared to lesser skilled athletes^49,50^, and studies have demonstrated a positive relationship between change in mental representation structure and longer-term adaptations to motor skill performance after movement simulation-based training^15,51^. Therefore, had the training been prolonged with data collected over a longer time period, it is expected that the improvements in perceptual cognitive scaffolding demonstrated at this early stage of motor learning after AOMI training would likely transfer to improved motor skill performance over time.

Despite the potential importance of our findings, this research is not without limitations. The main limitations of the study pertain to the complexity of the motor skill, the AOMI training schedule adopted. Research investigating the effects of AOMI training on motor skill performance has predominantly focused on simple, discrete motor tasks^22^. According to Guadagnoli and Lee’s challenge-point framework^52^, motor learning is facilitated when an optimal amount of information is afforded to the learner, and this is dependent on her/his skill level and the difficulty of the motor skill being learned. For the novice learners taking part in the current study, the high complexity of the Osoto Gari throw combined with the lack of feedback provided during training likely increased the cognitive processing demands placed on the novices during the AOMI training sessions, possibly inhibiting the motor learning process. This issue may have been exacerbated by the scheduling of the AOMI training utilized in this study. The AOMI training conditions required the novice learners to simultaneously engage with AO and MI of the Osoto Gari throw. A body of work has shown that alternating between AO and MI trials during AOMI training may improve motor skill performance to a greater extent than this simultaneous approach for novice performers (e.g., Refs.^26,27,47^). It is possible that alternating AO and MI trials reduces working memory demands compared to simultaneous AOMI during this initial stage of learning, where a novices’ working memory capacity is greatly limited^53^. Future studies should draw from findings in the motor learning literature (e.g., Refs.^44,52,54^) and adopt diverse methodological approaches to comprehensively examine the effectiveness of AOMI training for motor skill learning across different levels of expertise and types of motor skill.

Recent technological advances mean high-end virtual reality (VR) systems are becoming more portable, affordable, and both valid and reliable for utilization in sporting contexts, particularly in the teaching and learning of motor skills^55^. This offers the opportunity for AOMI interventions to create AO content that is more immersive, and potentially easier to control and manipulate^17,29^, compared to conventional methods such as video recordings and physical demonstrations. To-date, AOMI research has predominantly used real-world footage of individuals performing motor skill(s) as the visual component of the AOMI training interventions. Due to the physical constraints of manipulating visual perspective of video footage for a complex whole-body movement task, the present study adopted a 3D-modelled skeleton avatar that was digitally-generated using whole-body motion capture data for the AO component of the AOMI training. This may have influenced the effectiveness of the intervention, as models that resemble the viewer have been shown to increase corticospinal excitability^56^, which has been shown to predict observational learning^57^. Indeed, responses to the social validation questionnaire (see Table S2 in the supplementary results) indicated that participants did not believe the avatar used for either AOMI training condition represented their body or performance of the motor skill. A recent study employing digitally-generated avatars supported the importance of perceived avatar similarity, suggesting avatars that represent the learner provide attentional and motivational benefits towards the learning process^58^. We suggest future studies use immersive technologies such as VR to provide representative and controllable avatars in AOMI interventions, as these could provide similar benefits to real-life video modelling without the physical constraints noted when trying to manipulate visual perspective in this study^29,59^.

In summary, the present study examined the influence of AO perspective on the effectiveness of AOMI training for novices learning an Osoto Gari judo throw. In contrast to our predictions, there was little support for either type of AOMI training improving motor skill performance more than the Control training condition across the learning period. However, AOMI_ALLO_ training reduced error scores for final right hip flexion angle compared to AOMI_EGO_ and Control training, and peak right ankle velocity compared to AOMI_EGO_ training, suggesting specific benefits to the late phase of the movement that were not depicted in the AO content for the AOMI_EGO_ training condition. Self-efficacy levels also increased across all training conditions, with no significant differences reported for AOMI compared to Control training across time. Both AOMI_EGO_ and AOMI_ALLO_ training led to the development of more functional mental representation structures compared to Control training. This improvement in novices’ perceptual-cognitive scaffolding after a relatively short motor learning period is likely to transfer to improved motor skill performance if training is prolonged. Overall, the findings show mixed influence of AO perspective on the effectiveness of AOMI training for the learning measures adopted in this study, providing some support for the use of AOMI_ALLO_ training for novices learning complex, serial motor skills in sport.

## Methods

### Study design

The study was conducted in accordance with ethical guidelines and the study approval was granted from the University of Roehampton Ethical Committee. All study materials are stored as supplementary files on the Open Science Framework (https://osf.io/4fhnx). The study employed a motor learning design that incorporated a pre-test (Day 1), acquisition (Days 2–6), post-test (Day 7), and one-week delayed retention-test (Day 14). The between-subject factor was training condition (AOMI_EGO_ vs AOMI_ALLO_ vs Control) and the within-subject factor was test phase (pre-test vs post-test vs retention test). The dependent variables recorded to measure learning were motor skill performance, self-efficacy, and mental representation structures.

### Participants

Thirty right-handed participants (mean age = 27.2 ± 7.82 years) took part in the experiment. AOMI training that incorporates physical practice has a medium-large positive effect (*f* = 0.34) on movement outcomes compared to Control training conditions^22^. This effect size was input in to an a priori power analysis to determine the study sample size via G*Power (^60^; *F* tests, repeated measures, within-between interaction, for a Type I error probability of 0.05, a Type II error probability of 0.90^61^). A study sample of twenty-seven participants was required to achieve adequate power, but thirty participants were recruited to account for potential dropout (resultant *f* = 0.31). Participants were classified as novices in Brazilian jiu-jitsu and grappling sports (< 6 months experience) and received a £20 Amazon voucher as reimbursement for their involvement in the study. Participants were screened for imagery ability using the Vividness of Movement Imagery Questionnaire 2 (VMIQ-2^62^). VMIQ-2^62^ scores indicated that participants were able to generate moderately clear and vivid internal (23.90 ± 11.47), external (28.43 ± 11.47), and kinesthetic imagery (26.13 ± 14.12).

### Motor skill

The motor skill to be learned in this study was based on an Osoto Gari judo throw, where participants performed a shadow version of the movement without an opponent. The Osoto Gari throw is a complex full-body serial motor skill that consists of multiple components. First, the thrower grips the opponent behind the neck and on the inside of their arm to prepare to leverage them. Then, the thrower disrupts the balance of the opponent and steps through before swinging the unplanted leg forward and then backwards, sweeping the opponent’s leg to complete the throw. Finally, the thrower brings the unplanted leg forward and raises their body height to return to their starting position. For the shadow version of the Osoto Gari judo throw utilized in this study, participants visualised the opponent during their performance of the movement rather than physically performing it on an opponent. This use of shadow drills is commonplace within grappling sports such as wrestling and judo, particularly for beginners to help them learn the fundamentals of a movement sequence before progressing to drills with an opponent.

### Measures of learning

#### Motor skill performance

Motor skill performance was measured at all three test phases using biomechanical kinematic markers underpinning successful movements. 10 movement trials were collected at each test phase, with a total of 30 trials per participant. Three-dimensional marker positions were recorded using a 12 camera Vicon Vantage motion capture system (Vicon, Oxford, UK) sampling at 100Hz. Participants wore 37 reflective skin markers at selective anatomical landmarks according to the Vicon Plug-in gait marker set, which were tracked throughout all movement trials and filtered using a Butterworth fourth order low pass filter with a cut-off frequency of 6Hz, and then used to create a whole-body model. Eight discrete kinematic variables were extracted from each test trial of the shadow Osoto Gari movement: Initial Right Hip Flexion, Final Right Hip Flexion, Base of Support, Horizontal Centre of Mass, Vertical Centre of Mass, Peak Right Ankle Velocity, Peak Right Shoulder Velocity, and Peak Velocity Time Difference (detailed in Table 1). A skilled grappling athlete performed 10 successful trials of the shadow Osoto Gari movement and the mean score for each kinematic variable was used as a reference value. The absolute error score (i.e., difference between the participant’s recorded value and the reference value) was calculated using an average score of the participants 10 movement trials in each test phase, with a lower error score for each discrete kinematic measure representing more successful motor skill performance.

**Table 1:** Discrete kinematic measures recorded to represent motor skill performance for the Osoto Gari throw.

Discrete kinematic measure	Description	Interpretation	Determination
Initial and final right hip flexion (degrees)	Right hip flexion angle when the right ankle aligns with the left ankle (initial), and at the end of the swing (final), during the right leg swing back phase	Provides insight on the torso and upper leg acting as a single unit during the right leg swing back phase	Hip Flexion Angle is determined from the Vicon Plug-in Gait model where the angle between the pelvis and upper leg segments is calculated using the vector dot product
Base of support (%)	Horizontal distance between the right and left ankle (normalised to leg length) immediately prior to the initiation of the right leg swing phase	Provides insight on the stability of the body	Horizontal distance calculated between the right and left ankle marker and reported as a percentage of leg length
Horizontal centre of mass (%)	Horizontal position of the centre of mass within the base of support immediately prior to the initiation of the right leg swing phase	Provides insight on the stability of the body	Position of Centre of Mass is determined from the Vicon Plug-in Gait model and reported as a percentage of Base of Support
Vertical centre of mass (%)	Vertical position of the centre of mass within the base of support immediately prior to the initiation of the right leg swing phase	Provides insight on the height of the Centre of Mass from the ground	Position of Centre of Mass is determined from the Vicon Plug-in Gait model and reported as a percentage of leg length
Peak right ankle velocity (m/s)	Peak upward vertical velocity of the right ankle during the right leg swing back phase	Provides insight on lower body momentum being transferred to the upper body	Vertical Velocity is calculated from the linear displacement and time as determined from Vicon Plug-in Gait using first principle differentiation
Peak right shoulder velocity (m/s)	Peak downward vertical velocity of the right shoulder during the leg swing back phase	Provides insight on the momentum of the upper body	Vertical Velocity is calculated from the linear displacement and time as determined from Vicon Plug-in Gait using first principle differentiation
Peak velocity time difference (s)	Time difference between peak right ankle and right shoulder vertical velocity during the right leg swing back phase	Provides insight on the synchronisation between the upper and lower body	Temporal values are calculated from the frame rate of the Vicon system

#### self-efficacy

Self-efficacy was assessed using a bespoke 6-item self-report questionnaire developed using efficacy measurement guidelines^63^. The questionnaire was tailored for the shadow Osoto Gari movement and required participants to make confidence judgments about their ability to perform the different components of the movement, as well as the overall movement. Participants rated each item on an 11-point Likert scale from 0 (not at all confident) to 10 (completely confident). The specific components of the movement were taken from grappling resources (i.e., judo tutorials of the throw, as well as the first author’s own understanding of the movement as a skilled grappling athlete).

#### Mental representation structure

Mental representation structure was assessed using SDA-M as an indicator of accurate representation of the shadow Osoto Gari movement in long-term memory^42^. As per recommendations^64^, the basic action concepts (BACs) for the shadow Osoto Gari movement were initially developed by the research team using the above-mentioned grappling resources and first author knowledge. These were then rated by an expert panel including two skilled grappling athletes and two coaches with advanced knowledge and experience of performing/teaching the Osoto Gari throw. The final list of BACs (Table 2) was used for the SDA-M splitting procedure in this study. This involved one BAC being displayed on the screen (the anchor concept) while the participant decides if the other BACs (*n* = 10), which are displayed in a randomized order, are directly related to the anchor concept. As soon as all the decisions have been recorded for that anchor concept, the procedure repeats until all BACs have taken the anchor position and all decisions have been made. The whole split procedure lasted approximately 15–20 min for a total of 110 decisions (11 × 10). As skilled grappling athletes, the first author and a member of the expert panel completed the SDA-M splitting procedure to create a mental representation structure for the Osoto Gari as a reference point for this study (Fig. 1).

**Table 2:** Basic action concepts of the Osoto Gari throw.

Number	Basic action concept (BAC)
1	Left arm pulls partner's right arm forward and upward
2	Right arm pulls partner's neck towards yours
3	Left leg steps forward past partner's right leg
4	Contact is made with partner between their right hip and right upper body
5	Right leg swings through
6	Right leg swings back
7	Right shoulder drives forward and downwards into partner
8	Upper body tips forward and downward
9	Right leg lifts partner's right leg at the back of their knee
10	Bring upper body back upright
11	Bring right leg back to a secure stand

### Training conditions

#### Egocentric AOMI (AOMI_EGO_)

In the AOMI_EGO_ training condition, participants watched a 4-s video of an avatar performing the shadow Osoto Gari movement successfully from an egocentric perspective (Fig. 9a) and simultaneously imagined the kinesthetic sensations involved with performing the movement her/himself for each trial. The avatar represented the three-dimensional model from the motion capture of a skilled grappling athlete performing the shadow Osoto Gari movement. The avatar was used in order to manipulate perspective in a controlled and accurate manner for this complex whole-body movement. This permitted us to develop egocentric perspective video that provided a suitable amount of visual information of the shadow Osoto Gari movement for novice learners. Through pilot work using chest- and head-worn video cameras, we identified that it was not possible to develop usable real-world video footage from an egocentric perspective for this movement task. During each training session, the participant engaged with sixty AOMI_EGO_ trials broken down into six blocks of ten trials, totalling 240 s of video trials. After five consecutive AOMI_EGO_ trials, the participant performed five physical practice trials of the shadow Osoto Gari movement, meaning a total of sixty physical practice trials were completed alongside the video trials during every training session.

#### Allocentric AOMI (AOMI_ALLO_)

In the AOMI_ALLO_ training condition, the 4-s video displayed the avatar performing the shadow Osoto Gari movement from an allocentric perspective (Fig. 9b) in each trial. All other aspects of this training condition mirrored the protocol reported above for the AOMI_EGO_ training condition.

#### Control

Participants allocated to the Control training condition watched video extracts from an interview with a professional grappling athlete who talked about their general experiences as a competitor but did not include any technical information about the Osoto Gari judo throw. This is a common control condition used in the AOMI literature (e.g., Refs.^26,27^). During each training session, the participant watched six 40-s extracts from the recorded interview to match the total duration for sixty repetitions of the intervention footage used in the AOMI_EGO_ and AOMI_ALLO_ training conditions. After watching each 40-s extract, the participant performed ten physical practice trials of the shadow Osoto Gari movement, meaning a total of sixty physical practice trials were completed per training session.

### Procedure

#### Familiarization and pre-test phase

The participant arrived at the biomechanics laboratory at the first author’s university to complete the pre-test data collection (Day 1). Participants were initially briefed on the study requirements and provided informed consent, before being prepared for motion capture data collection. Participants were then shown a demonstration video that displayed a skilled grappling athlete performing four Osoto Gari throws on a partner in order to give participants a reference point for visualizing an opponent when performing the shadow movement without an opponent. Participants then practiced ten repetitions of the shadow movement without feedback, before completing the self-efficacy questionnaire and SDA-M procedure. To complete the pre-test phase, participants performed ten ‘test’ repetitions of the shadow Osoto Gari movement whilst kinematic data was collected.

#### Acquisition phase

All participants took part in five training sessions during the acquisition phase of the experiment (Days 2–6). For each session, participants were first led through a 5-min dynamic warm up that included all muscle groups used during the shadow Osoto Gari movement. Participants then watched the aforementioned demonstration video to re-familiarise with the different components of the throw when performed on another person, before receiving instructions based on their allocated training condition (see supplementary files for full instructions for each training condition) and engaged in the training protocol. For all training conditions, the participant completed a total of 20-min non-physical practice (i.e., engagement with the stimuli and task associated with their allocated training condition) and 300 physical practice trials without an opponent for the shadow Osoto Gari movement.

#### Post-test and retention-test phases

Participants returned to the biomechanics laboratory one day and eight days after finishing the final training session of the acquisition phase to complete the post-test (Day 7) and retention-test (Day 14) data collections, respectively. The protocol for both data collections mirrored that of the pre-test, recording movement kinematics, self-efficacy, and mental representation structures for the shadow Osoto Gari movement for a second and third instance across the study period. At the end of the experiment, participants allocated to the AOMI_EGO_ and AOMI_ALLO_ training conditions completed a social validation questionnaire and interview^65^. The social validation procedures aimed to gather perceived changes in motor skill performance, self-efficacy and mental representations for the training condition, and assess perceived ability to engage with the simulation processes and embody the movements displayed by the avatar during the AOMI training protocol (see Table 3 and Supplementary Material for full details and reporting of social validation data).

### Kinematic data extraction

#### Initial and final right hip flexion

During the right leg swing back phase, the torso and upper leg segments should act as a single unit, meaning the angle between the upper leg and pelvis (i.e., hip flexion angle) should not alter to enhance the transfer of momentum from the distal to the proximal segment. Two discrete variables of hip flexion angle were extracted during the right leg swing back phase (Fig. 10). For the ‘initial hip flexion’ angle (Fig. 10a), the measure was taken at the time where the right ankle aligns with the left ankle in the sagittal plane. For the ‘final hip flexion’ angle (Fig. 10b), the measure was taken at the time just prior to the torso moving vertically upwards at the end of the leg swing back phase.

#### Base of support

**Figure 11:**
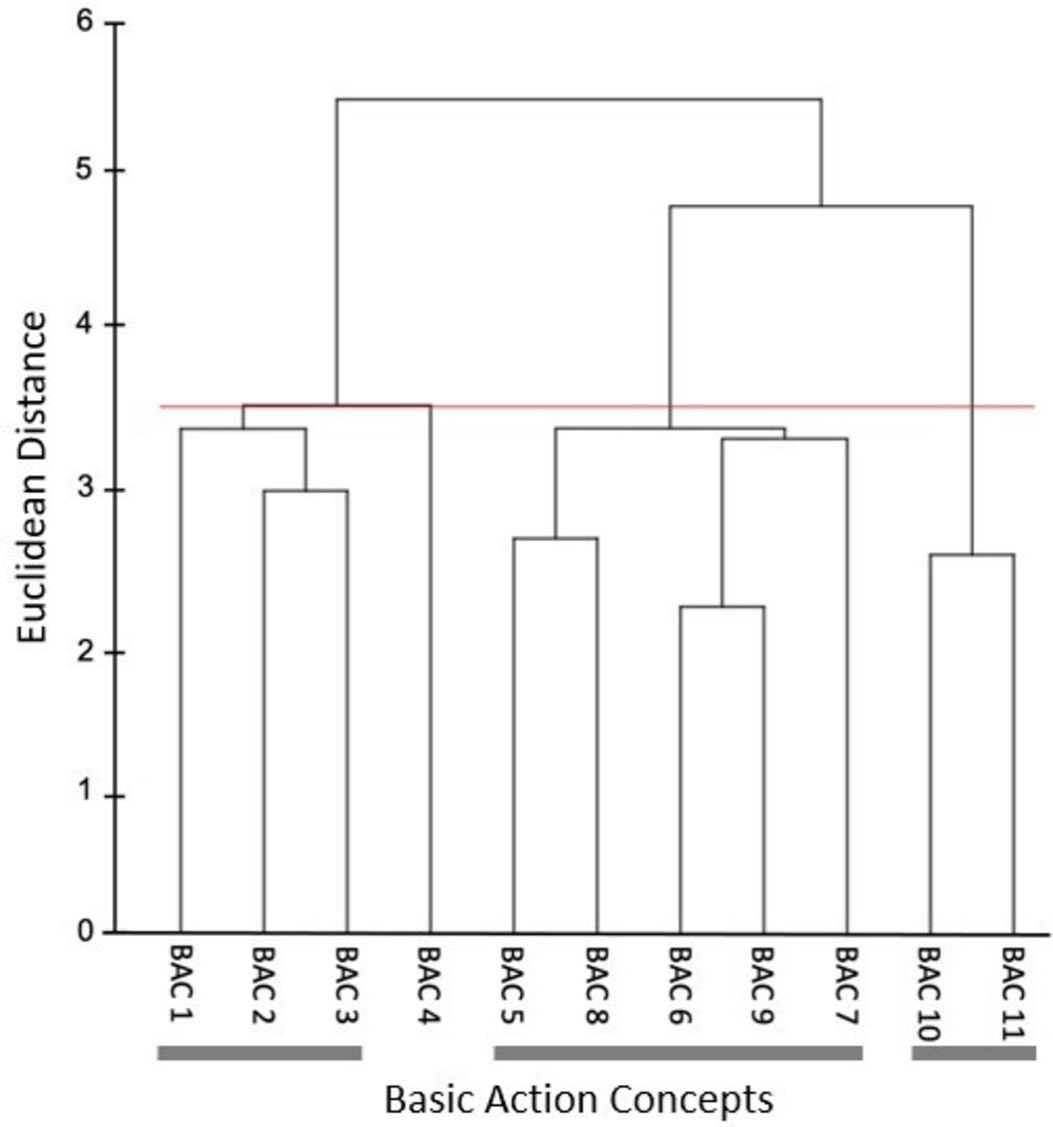
Mean group tree diagram of the Osoto Gari for the reference group of two skilled grappling athletes. Each BAC is labelled on the x-axis (for the list of BACs, see Table 2). The numbers on the y-axis display Euclidean distances. The lower the Euclidean distance between BACs, the closer the BACs are. The horizontal red line marks the critical value *d*_*crit*_ for a given *α*-level (*d*_*crit*_ = 3.41; *α* = 0.05). Thick horizontal grey lines below the BAC labels on the x-axis depict clusters of BACs.

It is important to have a stable base of support at the point of initiating the right leg swing phase to allow for the controlled production of force during the right leg swing. The horizontal distance between the right and left ankle was extracted at the point of the left ankle stepping prior to the swinging motion of the right leg (Fig. 11). At this point, the horizontal distance was calculated between the right and left ankle marker and normalized as a percentage of the participant’s leg length.

#### Horizontal and vertical centre of mass

**Figure 12:**
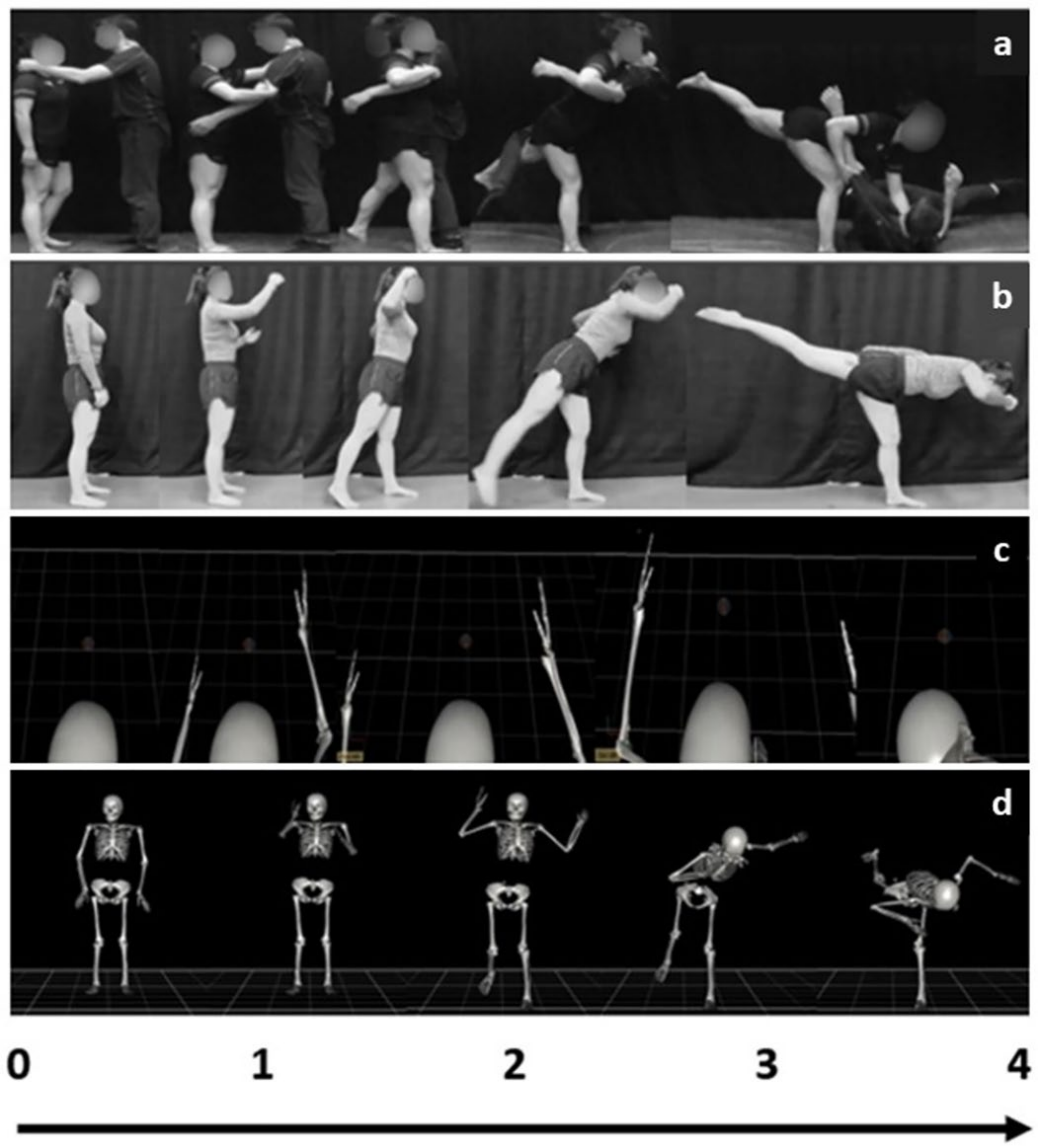
A visual depiction of (**a**) The demonstration Osoto Gari movement with an opponent, (**b**) the shadow Osoto Gari movement task performed in this study, and the avatar video footage adopted in the (**c**) AOMI_EGO_ and (**d**) AOMI_ALLO_ training conditions.

It is important the participant maintains postural control and stability at the point of initiating the right leg swing phase to allow for the controlled production of force during the right leg swing. The positions of the centre of mass in the horizontal and vertical direction were recorded at the point when the base of support is set (Fig. 12). Horizontal position of the centre of mass was normalized as a percentage of the absolute width of the base of support measure, and the vertical position of the centre of mass was normalized as a percentage of leg length. Both measures provided insight on the stability of the body in the sagittal plane.

#### Peak right ankle velocity, peak right shoulder velocity, and peak velocity time difference

**Figure 13:**
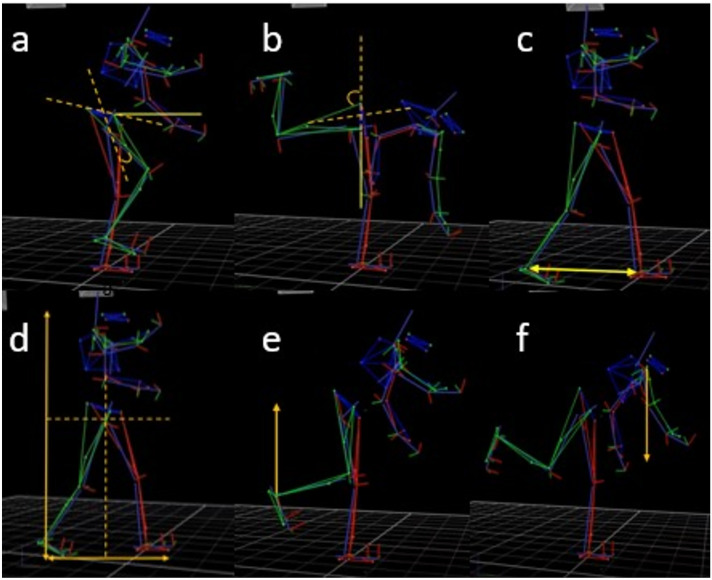
Kinematic models of the data extraction points for biomechanical kinematic markers underpinning successful movements as a measure of motor skill performance in this study. Panels display extraction points for the (**a**) initial hip flexion angle during the right leg swing back phase, (**b**) end hip flexion angle during the right leg swing back phase, (**c**) base of support prior to initiation of the right leg swing phase, (**d**) horizontal and vertical centre of mass prior to initiation of the right leg swing phase, (**e**) peak velocity of the right ankle during the right leg swing phase, and (**b**) peak velocity of the right shoulder during the right leg swing phase.

It is important the participant achieves maximum velocity in a controlled manner during the right leg swing back phase as this component of the movement aims to throw a potential opponent to the floor, as visualised during the shadow Osoto Gari movement. The peak vertical velocity of the right ankle (Fig. 13a) provides insight on the momentum of the lower body being transferred to the upper body during the right leg swing back phase. The peak vertical velocity of the right shoulder (Fig. 13b) provides insight on the momentum of the upper body during the right leg swing back phase. Both velocity measures were extracted as the maximum vertical velocity reported for each joint during the right leg swing back phase. The peak velocity time difference measure provides insight on the synchronization between the upper and lower body during the right leg swing back phase. This was taken as the time difference between the points of the leg swing back phase that peak ankle velocity and peak shoulder velocity occurred.

### Data analysis

#### Motor skill performance and self-efficacy

Multi-level linear models (MLM) were run for motor skill performance and self-efficacy data using the ‘lme4’ package^66^ in R studio statistical software (version 4.2.1). Significance was calculated using the ‘lmerTest’ package^67^, which applies Satterthwaite’s method to estimate degrees of freedom and generate *p*-values for mixed models. Mean overall self-efficacy scores and error scores for each discrete kinematic measure served as separate dependent variables, and ‘participant’ was included as a random intercept. We attempted to model random slopes to account for individual differences in response across test phases, but the model would not run because it lacked sufficient data to reliably estimate all the specified random effects parameters. Outliers were identified for motor skill performance and self-efficacy data using and would not run because it lacked sufficient data to reliably estimate all the specified random effects interquartile range values. Twenty-three individual data points were removed as outliers across the nine MLM analyses, as indicated by black markers on Figs. 2, 9, 10, 11, 12, 13.

To check for any potential effects of imagery ability on the respective scores^68^ a second identical model was run with VMIQ-2^62^ kinesthetic imagery scores added as a covariate for each of the dependent variables. The MLM’s incorporating imagery ability as a co-variate decreased the accuracy of the models compared to the original models, and imagery ability scores did not significantly influence error scores for the eight discrete kinematic measures of motor skill performance, or self-efficacy scores (see Supplementary file for reporting of model accuracy and influence statistics for each secondary MLM). A two-step approach was followed to account for potential washout effects in the motor skill performance data that may have resulted from the large trial-count used across the test phases. First, we re-ran the primary analyses for the eight discrete kinematic measures of motor skill performance using mean error scores from the first 3 trials, rather than the full 10 trials. We saw a similar profile of data and noted no differences in main or interaction effects for any of the discrete kinematic measures when compared to the full dataset. We then created data sheets that included raw error scores for each individual trial and loaded ‘Trial Number’ as a factor into the MLMs as a continuous fixed effect. We found no significant effect of Trial Number for any of the variables across the three training conditions and test phases, indicating changes in performance of the motor skill were not washed out over the course of the test phase trials.

#### Mental representation structure

Drawing on the Euclidean distance scaling between BACs as obtained by the SDA-M split procedure, cluster analyses (*α* = 0.5, *d*_*crit*_ = 3.99) were performed to outline the structure of mental representations. Mean group tree diagrams were computed for each experimental condition (AOMI_EGO_, AOMI_ALLO,_ Control) at each test phase (Pre-test, Post-test, retention-test). Analysis of invariance was conducted to compare the different cluster solutions between training conditions and across test phases. Two cluster solutions are variant when *λ* < 0.68, and are invariant when cluster solutions are *λ* ≥ 0.68^64^. Closer proximity with the reference structure indicates a more functionally accurate representation of the Osoto Gari. The Adjusted Rand Index (ARI^69^) was calculated as a similarity metric between the structures for the training conditions and the reference structure at each test phase. ARI values between -1 (structures are different) and 1 (structures are the same) were obtained, with a greater positive difference in ARI values between pre-test and retention-test indicating greater learning of the cognitive aspects of the Osoto Gari.

### Supplementary Information

Supplementary Information.

## Data Availability

The datasets generated during and/or analysed during the current study are publicly available on the Open Science Framework and can be accessed using the following web link: (https://osf.io/4fhnx).
