# Supplementary material for: Action observation perspective influences the effectiveness of combined action observation and motor imagery training for novices learning an Osoto Gari judo throw
Source: Sci Rep. 2024 Aug 28;14:19990. doi: 10.1038/s41598-024-70315-8 (PMC11358477; doi:10.1038/s41598-024-70315-8)
Supplement: Supplementary file 1 — Supplementary Information. [file 41598_2024_70315_MOESM1_ESM.pdf]

Action observation perspective influences the effectiveness of combined action observation and motor imagery training for novices learning an Osoto Gari judo throw

Samantha Chye[1†], Ashika Chembila Valappil[1], Ryan Knight[1], Andrew Greene[1], David Shearer[2], Cornelia Frank[3], Ceri Diss[1], & Adam Bruton[1,4*]

Author Note: Samantha Chye is the first author for this manuscript. *Adam Bruton is the corresponding author for this manuscript: adam.bruton@brunel.ac.uk, HNZW 271, Heinz Wolff Building, Brunel University London, Uxbridge, UB8 3PH, United Kingdom

[1] School of Life and Health Sciences, University of Roehampton, United Kingdom
[2] Faculty of Life Sciences and Education, University of South Wales, United Kingdom
[3] Department of Sports and Movement Science, Osnabrück University, Germany
[4] Department of Life Sciences, Brunel University London, United Kingdom, Email: adam.bruton@brunel.ac.uk

Supplementary Results

Table S1. Random effect residuals for 'participant' for each kinematic variable and the amount of variance accounted for in each variable.

Variable	τ00 Participant	SD	Variance
Initial Right Hip Flexion	34.54	5.88	53.9%
Final Right Hip Flexion	125.1	11.19	64.3%
Base of Support	163	12.77	60.3%
Horizontal Centre of Mass	17.85	4.23	53.5%
Vertical Centre of Mass	8.74	2.96	62.83%
Peak Right Ankle Velocity	1.13	1.06	77.26%
Peak Right Shoulder Velocity	0.17	0.41	65%
Peak Velocity Time Difference	0.01	0.07	55.6%
Self-Efficacy Score	1.49	1.22	71%

Exploring Imagery Ability as a Co-Variate

Motor Skill Performance

Initial Right Hip Flexion. The MLM incorporating imagery ability as a co-variate decreased the accuracy of the model (*BIC* = 638.4) compared to the original model (*BIC* = 634.9), and imagery ability scores did not significantly influence initial right hip flexion error scores (*ß* = 0.05, *p* = .33).

Final Right Hip Flexion. The MLM incorporating imagery ability as a co-variate decreased the accuracy of the model (*BIC* = 707.1) compared to the original model (*BIC* = 704.5), and imagery ability scores did not significantly influence final right hip flexion error scores (*ß* = -0.13, *p* = .19).

Base of Support. The MLM incorporating imagery ability as a co-variate decreased the accuracy of the model (*BIC* = 772.5) compared to the original model (*BIC* = 769.3), and imagery ability scores did not significantly influence base of support error scores (*ß* = -0.13, *p* = .27).

Horizontal Centre of Mass. The MLM incorporating imagery ability as a co-variate decreased the accuracy of the model (*BIC* = 575.6) compared to the original model (*BIC* = 571.9), and imagery ability scores did not significantly influence horizontal centre of mass error scores (*ß* = 0.03, *p* = .38).

Vertical Centre of Mass. The MLM incorporating imagery ability as a co-variate decreased the accuracy of the model (*BIC* = 511.4) compared to the original model (*BIC* = 506.9), and imagery ability scores did not significantly influence vertical centre of mass error scores (*ß* = -0.004, *p* = .89).

Peak Right Ankle Velocity. The MLM incorporating imagery ability as a co-variate decreased the accuracy of the model (*BIC* = 282.1) compared to the original model (*BIC* = 278.3), and imagery ability scores did not significantly influence peak right ankle velocity error scores (*ß* = -0.008, *p* = .42).

Peak Right Shoulder Velocity. The MLM incorporating imagery ability as a co-variate decreased the accuracy of the model (*BIC* = 144.8) compared to the original model (*BIC* = 141.1), and imagery ability scores did not significantly influence peak right shoulder velocity error scores (*ß* = -0.003, *p* = .37).

Peak Velocity Time Difference. The MLM incorporating imagery ability as a co-variate decreased the accuracy of the model (*BIC* = -127.9) compared to the original model (*BIC* = -131.2), and imagery ability scores did not significantly influence peak velocity time difference error scores (*ß* = -0.001, *p* = .30).

Self-Efficacy

The MLM incorporating imagery ability as a co-variate decreased the accuracy of the model (*BIC* = 321.7) compared to the original model (*BIC* = 317.9), and imagery ability scores did not significantly influence self-efficacy scores ($ß = 0.01$, $p = .43$).

Social Validation Data

Participant's allocated to the two AOMI training groups responded positively to the social validation questionnaire (Table 3) with a mean rating of 0.89 recorded for the $AOMI_{ALLO}$ training condition and 1.07 recorded for the $AOMI_{EGO}$ training condition. Avatar representativeness reflected the only significant difference between the two training conditions, with participants reporting that the avatar displayed in the videos used in the $AOMI_{EGO}$ training to be more representative of their own movements than the avatar displayed in the $AOMI_{ALLO}$ training videos. Below are summary responses and representative quotes for the effects of the different AOMI training conditions on the measures of learning.

Motor Skill Performance

Nine participants (90%) believed their motor skill performance improved after both $AOMI_{EGO}$ and $AOMI_{ALLO}$ training. The participants that perceived $AOMI_{EGO}$ training as performance enhancing suggested that the AOMI stimuli promoted a positive sense of self and the videos included key triggers that helped their imagery and performance of the Osoto Gari; *P15 "It allowed me to kind of see more of what the movement entailed and especially from the 1st person perspective it let me kind of imagine myself doing it instead of just watching someone else do it".* The participants that perceived $AOMI_{ALLO}$ training as performance enhancing suggested the video provided useful visual information and acted as a positive reference point for their own performances of the Osoto Gari; *P6 "Being able to*

repeatedly reference a stable example allowed for corrections with each iteration I was doing to correct and have that reference".

Self-Efficacy

Nine participants (90%) believed their self-efficacy increased after $AOMI_{EGO}$ and seven participants (70%) believed their self-efficacy increased after $AOMI_{ALLO}$ training. The participants that perceived $AOMI_{EGO}$ training to have a positive impact on their self-efficacy suggested that the videos facilitated their imagery of the Osoto Gari, primarily enhancing their expectations through provision of positive mastery experiences; *P16 "Because I was seeing it happen on a screen but then I could also visualize myself doing it like I knew how the certain movements would go about so it was quite easy to see me doing it, it was making me more confident in that".* The participants that perceived $AOMI_{ALLO}$ training increased their confidence in performing the Osoto Gari mostly attributed this to the video showing that the throw can be performed successfully (positive vicarious experiences); *P9 "Because the skeleton video made it look easier and so I thought, well, I'm going to try to repeat this as it seems very possible and easy enough".*

Mental Representation Structure

Eight participants (80%) believed their understanding of the movement improved after both $AOMI_{EGO}$ and $AOMI_{ALLO}$ training. The participants that perceived $AOMI_{EGO}$ training to advance their knowledge about the Osoto Gari suggested it provided key sensory cues that they utilized to consolidate their understanding of the movement sequencing; *P12 "every action or movement started to make sense to me in the term of succession and therefore having a clear understanding of the whole movement and the sensation".* The participants that perceived $AOMI_{ALLO}$ training as helpful when retaining the Osoto Gari movement information suggested watching (AO) the model drove any benefits; *P18*

"Because of watching it multiple times and repeating it and the information just stuck in my head".

Table S2. Social validation questionnaire responses for the AOMI training conditions.

Social Validation Questionnaire Item	$AOMI_{EGO}$ *n = 10*	$AOMI_{ALLO}$ *n = 10*	Diff *t-statistic*
**Motor Skill Performance[a] –** perceived impact of AOMI training on performance of Osoto Gari.	2.2 ± 1.1	2.5 ± 1.6	0.5
**Self-Efficacy[a] -** perceived impact of AOMI training on confidence to perform Osoto Gari successfully.	2.5 ± 1.4	1.8 ± 1.8	-1.0
**Mental Representation Structure[a] -** perceived impact of AOMI training on understanding of the Osoto Gari.	2.4 ± 1.5	2.1 ± 1.8	-0.4
**AO Ability[b] –** ability to watch the Osoto Gari videos during the AOMI training.	1.0 ± 1.4	1.2 ± 1.4	0.3
**MI Ability[b] -** ability to imagine the sensations involved with the Osoto Gari during the AOMI training.	0.8 ± 1.6	1.0 ± 1.5	0.3
**AOMI Difficulty[b] -** ability to simultaneously watch the videos and imagine the sensations involved with the Osoto Gari during the AOMI training.	-0.3 ± 1.7	-0.1 ± 1.4	0.3
**MI Vividness[c] –** clarity and vividness of the sensations generated when imagining the Osoto Gari during the AOMI training.	0.1 ± 1.5	0.4 ± 1.3	0.5
**Avatar Representativeness[d] –** perceived similarity between the participant's Osoto Gari and the avatar's performance in the videos during AOMI training.	0.9 ± 1.2	-0.5 ± 1.3	-2.5*
**Avatar Plausibility[d] –** perceived plausibility of the Osoto Gari performed by the avatar in the videos during AOMI training.	1.5 ± 1.2	1 ± 1.6	-0.8
**Avatar Ownership[d] -** perceived ownership of the Osoto Gari performed by the avatar in the videos during AOMI training.	-0.4 ± 1.8	-0.5 ± 1.4	-0.1

*Note. The following rating scales were adopted across the questionnaire: [a] -5 (negative effect) to 5 (positive effect); [b] -3 (very difficult) to 3 (very easy); [c] -3 (very unclear and not at all vivid) to 3 (very clear and vivid); [d] -3 (strongly disagree) to 3 (strongly agree). *p < .05*
